# Untargeted Metabolomics Used to Describe the Chemical Composition, Antioxidant and Antimicrobial Effects of Extracts from *Pleurotus* spp. Mycelium Grown in Different Culture Media

**DOI:** 10.3390/antibiotics11111468

**Published:** 2022-10-25

**Authors:** Giancarlo Angeles Flores, Carolina Elena Girometta, Gaia Cusumano, Paola Angelini, Bruno Tirillini, Federica Ianni, Francesca Blasi, Lina Cossignani, Roberto Maria Pellegrino, Carla Emiliani, Roberto Venanzoni, Giuseppe Venturella, Pasqualina Colasuonno, Fortunato Cirlincione, Maria Letizia Gargano, Gokhan Zengin, Alessandra Acquaviva, Simonetta Cristina Di Simone, Giustino Orlando, Luigi Menghini, Claudio Ferrante

**Affiliations:** 1 Department of Chemistry, Biology and Biotechnology, University of Perugia, 06122 Perugia, Italy; 2 Department of Earth and Environmental Sciences (DSTA), University of Pavia, 27100 Pavia, Italy; 3 Department of Biomolecular Sciences, University of Urbino, 61029 Urbino, Italy; 4 Department of Pharmaceutical Sciences, University of Perugia, 06126 Perugia, Italy; 5 Center for Perinatal and Reproductive Medicine, Santa Maria della Misericordia University Hospital, University of Perugia, 06132 Perugia, Italy; 6 Department of Agricultural, Food and Forest Sciences, University of Palermo, Viale delle Scienze, Bldg. 5, 90128 Palermo, Italy; 7 Department of Agricultural and Environmental Science, University of Bari Aldo Moro, Via Amendola 165/A, 70126 Bari, Italy; 8 Physiology and Biochemistry Research Laboratory, Department of Biology, Science Faculty, Selcuk University, 42130 Konya, Turkey; 9 Botanic Garden “Giardino dei Semplici”, Department of Pharmacy, “Gabriele d’Annunzio” University, Via dei Vestini 31, 66100 Chieti, Italy

**Keywords:** *Pleurotus species*, metabolomics, antimicrobial effect, phenolic compounds

## Abstract

*Pleurotus* species isolated in vitro were studied to determine the effect of different media on their production of secondary metabolites, antimicrobial, and antioxidant activity. The different metabolites among *Pleurotus* samples covered a total of 58 pathways. Comparisons were made between the metabolic profiles of *Pleurotus* spp. mycelia grown in two substrates: Potato-dextrose-agar-PDA, used as control (S1), and PDA enriched with 0.5 % of wheat straw (S2). The main finding was that the metabolic pathways are strongly influenced by the chemical composition of the growth substrate. The antibacterial effects were particularly evident against *Escherichia coli*, whereas *Arthroderma curreyi* (CCF 5207) and *Trichophyton rubrum* (CCF 4933) were the dermatophytes more sensitive to the mushroom extracts. The present study supports more in-depth investigations, aimed at evaluating the influence of growth substrate on *Pleurotus* spp. antimicrobial and antioxidant properties.

## 1. Introduction

The genus *Pleurotus* (Fr.) P. Kumm. (Pleurotaceae, Basidiomycota) includes some of the main cultivated edible mushrooms in the world due to their gastronomic, nutritional, and medicinal properties, such as anti-inflammatory [1], antioxidant [2,3], antidiabetic [4], antitumor [5], and immunomodulating [6]. Due to their ability to improve protein content and quality, as well as the valuable health benefits of myco-chemicals or bioactive components present in these mushrooms [7,8,9], *Pleurotus* spp. can also be used to supplement different processed products such as bread and dairy foods. *Pleurotus* genus includes white rot fungi distributed all over the globe [10] due to their capacity to colonize and use accessible lignocellulosic materials and wastes, that have been considered suitable for bioconversion of agro-wastes into food and feed in developing countries [11,12,13,14,15]. They play an important role in managing organic waste whose disposal is problematic, e.g., those deriving from olive-oil production (i.e., olive pruning residues, olive mill wastes, and olive leaves) and wineries (e.g., grape marc) [16,17]

Compared to the most commonly cultivated mushrooms, such as *Agaricus bisporus*, *Pleurotus* species have the advantage of very simple cultivation; in fact, it could be sufficient to use a non-composted straw, chopped and soaked in water [18]. Based on the CABI Index Fungorum (http://www.indexfungorum.org/names/Names.asp; accessed 6 February 2022), the genus *Pleurotus* currently consists of about 217 accepted species all of which are edible and appreciated for their taste, aroma, and texture, as well as the health-enhancing bioactive potentials [19]. *P. ostreatus* is the most popular among *Pleurotus* mushrooms. It is a cosmopolitan species growing on dead wood of many broad-leaved and coniferous trees. Its cultivation is widespread throughout the world on a remarkable spectrum of lignocellulosic substrates such as maize straw, maize cob, palm kernel cake, sawdust, spent grain, rice bran, cereal grasses, sugarcane bagasse, coffee residues, coir waste, and cardboard industrial waste, etc. [15]. 

Much of the research on *Pleurotus* species has to date focused on the potential of various lignocellulosic by-products (e.g., cereal grasses, sugarcane bagasse, coffee residues, coir waste, and cardboard industrial waste) to support satisfactory mushroom yields [20,21,22], extraction of secondary metabolites (alkaloids, flavonoids, betalain among others) and pharmaceutical, biotechnological and food applications [23,24].

However, there is some research concerning the composition of fruiting bodies cultivated on various agro-wastes, and no data exist on the effect of cultural media on the mycelium content in bioactive compounds and their functional properties. The growth of mycelium is strongly influenced by many factors such as culture media, temperature, carbon and nitrogen sources, grain sources, and sources of lignocellulosic substrate [25,26]. Manipulating mycelium growth conditions is a common strategy used by pharmaceutical companies to improve the diversity of secondary metabolites of therapeutic interest [27]. 

The main objectives of the present work were (a) to investigate the suitability of two different cultural media for mycelium growth, and (b) to investigate how the culture medium affects the antimicrobial and antioxidant activity in the mycelial stage. Mass spectrometry (MS)-based metabolomic profiles coupled with multivariate statistical analysis will help determine the effects of different culture media as a function of metabolomic and transcriptomic disparity in *Pleurotus* spp. mycelia [28]. Additionally, the quantitative analysis of phenolic compounds has been carried out, as well.

## 2. Materials and Methods

### 2.1. Mushroom Material

The fruiting bodies of *P. columbinus*, *P. ostreatus*, *P. nebrodensis*, and *P. eryngii* species (*P. eryngii* var. *thapsiae*, *P. eryngii* var. *ferulae*, *P. eryngii* var. *elaeoselini*) were collected on different dates and in different locations (Table 1) and immediately transferred to the laboratory to obtain pure mycelial cultures.

Briefly, for the isolation of mycelia, context pieces (5 × 5 × 5 mm) were excised aseptically from the context of fresh Basidiomycota and transferred to Petri dishes containing Rose Bengal Chloramphenicol agar (Sigma-Aldrich, Milan, Italy) under sterile conditions. Petri dishes inoculated with 3–4 explants were then incubated at 25 °C in the dark for 7 days. 

Basidiomata identification was carried out by morphological and molecular analysis [29,30] in consideration of the available data on the occurrence of *Pleurotus* species in Italy [31].

### 2.2. Molecular Identification

To test the morphological identification (Table 1), the ITS region of the nrDNA was used as a fingerprint marker, as suggested for the wide majority of fungi [32] and successfully applied in previous works by the authors themselves [33,34,35]

The total genomic DNA was extracted using the ZR Fungal/Bacterial DNA Kit (Euroclone S.p.A., Milan, Italy). The genomic DNA quality and quantity were evaluated with BIORAD (Milan, Italy) model 200/2.0 Power Supply gel electrophoresis [0.8% agarose gel in 1× TBE buffer (89 mM Tris, 89 mM boric acid, 2 mM EDTA, pH 7.6)] in the presence of SafeView Nucleic Acid Stain (NBS Biologicals, Huntingdon, UK) and a MassRuler DNA Ladder Mix (Thermo Scientific, Vilnius, Lithuania), and visualized with Safe ImagerTM 2.0 Blue Light Trans illuminator Invitrogen (Parma, Italy). DNA samples were subsequently diluted with up to 10 μg/μL nuclease-free water before PCR amplification. The internal transcribed spacer (ITS) region of the nrDNA was amplified by ITS1F and ITS4 primers. SimpliAmp Thermal Cycler Applied Biosystems (Monza, Italy) was programmed as follows: one cycle of denaturation at 95 °C for 2.5 min; 35 cycles of denaturation at 95 °C for 20 s, annealing at 55 °C for 20 s and extension at 72 °C for 45 s; one final extension cycle at 72 °C for 7 min. Electrophoresis of PCR amplicons was carried out on 1.2% agarose gel as described above. The PCR amplified ITS fragment was purified using the ExoSapIT PCR Cleanup reagent (Thermo Fisher, Monza, Italy) and then sequenced by Macrogen Europe (Netherlands) (Table 2). 

### 2.3. Preparation of Samples

The in vitro culture of *Pleurotus* spp. was performed in the following solid media: (1) Potato-dextrose-agar-PDA, used as control (S1), and (2) PDA enriched with 0.5% of wheat straw (S2). Each medium in flasks was autoclaved at 121 °C for 20 min and subsequently dispensed into 15 100 mm Petri dishes. The mycelium discs (1 cm diameter) of each *Pleurotus* mushroom were placed in Petri dishes containing each culture medium (20 mL) under aseptic condition and incubated at 25 °C in the darkness. After 15 days of growth (when mycelium reached maximum radial growth in the PDA medium) the mycelium was recovered from the medium. All samples, realized in duplicate, were lyophilized (FreeZone 4.5 model 7750031, Labconco, Kansas, MO, USA), quantified, and reduced to a fine-dried powder (Supplementary Material: Tables S1 and S2). Preparation of mycelia extract: the lyophilized mycelia were extracted for 30 min with distilled and deionized water under ultrasonic agitation.

### 2.4. Untargeted LC-MS/MS-Based Metabolomics and Statistical Analysis

Untargeted LC/MS QTOF analysis was performed using a 1260 Infinity II LC System coupled with an Agilent 6530 Q-TOF spectrometer (Agilent Technologies, Santa Clara, CA USA). The LC consists of a quaternary pump, a thermostated column compartment, and an autosampler. Separation was carried out on an Agilent InfinityLab Poroshell 120 HILIC-Z, 2.1 × 150 mm, 2.7 µm at 25 °C, and 0.25 mL/min flow. The mobile phase consisted of a mixture of water (A) and water/ACN 15:85 (B) both containing a concentration of 10 mM ammonium acetate. Gradient was: time 0–3 min isocratic at A 2%, B 98%; time from 3 to 11 min: linear-gradient to A 30%, B 70; time 11–12 min linear gradient to A 60%, B 40%; time from 12 to 16 min: linear-gradient to A 95%, B 5%; time 16–18 min isocratic at A 95%, B 5%; time 18 min: stop run.

Spectrometric data were acquired in the 40–1700 m/z range both in negative and positive polarity. The Agilent JetStream source operated as follows: Gas Temp (N2) 200 °C, Drying Gas 10 L/min, Nebulizer 50 psi, Sheath Gas temp: 300 °C at 12 L/min.

Raw data were processed using MS-DIAL software (4.48) [36] to perform peak-picking, alignment, and peak integration. The MS signal threshold was set at 1000 counts. In the end, a data matrix was obtained reporting the accurate mass and area of each peak revealed in each sample analyzed.

The putative annotation of metabolites and the prediction of metabolic pathways was performed using the mummichog algorithm [37], implemented in the ‘MS Peaks to Pathways’ module of Metaboanalyst 5.0 [38]. It considers any possible adducts and different ionic polarities and classifies the peaks annotated on the basis of the *t*-test. In this case, the list of putative compounds was mapped onto the KEGG library of Saccaromices cerevisiae. ANOVA and Functional Meta-Analysis were also performed with MetaboAnalyst. For statistical analysis, samples were normalized by the median, followed by pareto scaling.

### 2.5. HPLC-DAD-MS Determination of Phenolic Compounds

The HPLC apparatus consisted of two PU-2080 PLUS chromatographic pumps, a DG-2080-54 line degasser, a mix-2080-32 mixer, UV, diode array (DAD) and detectors, a mass spectrometer (MS) detector (expression compact mass spectrometer), Advion, Ithaca, NY 14850, USA), an AS-2057 PLUS autosampler, and a CO-2060 PLUS column thermostat (all from Jasco, Tokyo, Japan). Integration was performed by ChromNAV2 Chromatography software. Before the injection in the HPLC apparatus, the extracts were centrifuged at 3500× *g* for 15 min, and the supernatant was diluted to 20 mg/mL. The extracts were analyzed for phenol quantitative determination using a reversed-phase HPLC-DAD-MS in gradient elution mode (Table 3). The separation was conducted within 60 mins of the chromatographic run, starting from the following separation conditions: 95% water with 0.1% formic acid, and 5% methanol with 0.1% formic acid (Table 4). The separation was performed on an Infinity lab Poroshell 120-SB reverse phase column (C18, 150 × 4.6 mm i.d., 2.7 μm) (Agilent, Santa Clara, CA, USA). The column temperature was set at 30 °C. Quantitative determination of phenolic compounds was performed via a DAD detector, at 254 nm. Quantification was done through 7-point calibration curves, with linearity coefficients (R^2^) > 0.999, in the concentration range of 2–140 µg/mL. The limits of detection were lower than 1 µg/mL for all assayed analytes. The area under the curve from HPLC chromatograms was used to quantify the analyte concentrations in the extracts. The extracts were also qualitatively analyzed with an MS detector in negative ion mode. MS signal identification was realized through comparison with a standard solution and MS spectra present in the MassBank Europe database.

### 2.6. Scavenging Effects

The scavenging effect of mushroom extracts on DPPH and ABTS radicals was evaluated as previously reported [33].

### 2.7. Antimicrobial Effects

The in vitro antimicrobial activity of extracts was assessed against the following Gram-negative and Gram-positive bacterial strains: *Escherichia coli* (ATCC 10536), *E. coli* (PeruMycA 2), *E. coli* (PeruMycA 3), *Bacillus cereus* (PeruMycA 4), *B. subtilis* (PeruMyc 6), *Salmonella typhy* (PeruMyc 7), *Pseudomonas aeruginosa* (ATCC 15442), and *Staphylococcus aureus* (ATCC 6538). Furthermore, the same extracts were assayed for the antifungal assays against different yeasts, dermatophyte, and fungal pool species: *Candida albicans* (YEPGA 6183), *C. tropicalis* (YEPGA 6184), *C. albicans* (YEPGA 6379), *C. parapsilopsis* (YEPGA 6551), Arthroderma crocatum (CCF 5300), *A. curreyi* (CCF 5207), *A. gypseum* (CCF 6261), *A. quadrifidum* (CCF 5792), *A. insingulare* (CCF 5417), A. quadrifidum (CCF 5792), *Trichophyton mentagrophytes* (CCF 4823), *T. mentagrophytes* (CCF 5930), *T. rubrum* (CCF 4933), and *T. tonsurans* (CCF 4834). Details are reported in our previous paper [2].

## 3. Results and Discussion

### 3.1. Mushroom Identification

The exact characterization and identification of medicinal mushrooms were fundamental for exploiting their full potential in the food and pharmaceutical industries [18].

The morphological characteristics of *Pleurotus* spp. (Table S1) fruiting bodies corresponded to those reported in the literature [29].

The taxonomic affiliation of the mushroom strains was performed by targeting the ITS region of the ribosomal DNA. Additionally, a BLAST search confirmed that our samples belong to *P. columbinus*, *P. eryngii* var. *thapsiae*, *P. ostreatus*, *P. nebrodensis*, *P. eryngii* var. *ferulae*, and *P. eryngii* var. *elaeoselini*, as it showed a close match with deposited sequences of these species. 

### 3.2. Untargeted LC-MS/MS-Based Metabolomics

In this study, the metabolomic profile of *Pleurotus* spp. was evaluated through mass spectrometry ultra-performance liquid chromatography-mass spectrometry (UHPLC)-QTOF method. The different metabolites in all *Pleurotus* samples covered a total of 58 pathways, including biotin metabolism, pantothenate and CoA biosynthesis, tryptophan metabolism, arginine biosynthesis, valine, leucine and isoleucine degradation, glutathione metabolism, one carbon pool by folate, vitamin B6 metabolism, sulfur metabolism, and riboflavin metabolism (Table 5).

**Figure 1:**
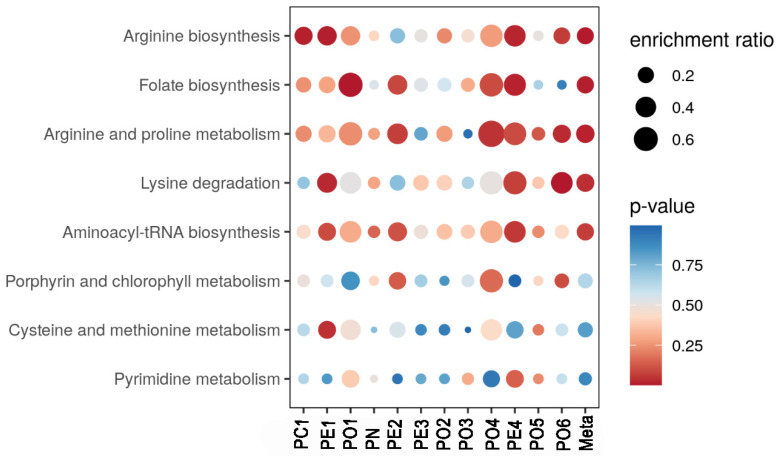
Comparative investigation for exploring the effect of different substrates on the metabolic profile was made between *Pleurotus* spp. mycelia grown in substrates S2. Substrate S1 was taken as a reference and data were calculated as mean differences compared to S1 (calibrator of the relative quantification). In the figure, red indicates a higher probability of metabolic pathway activation, whereas blue suggests a minor one.

**Figure 2:**
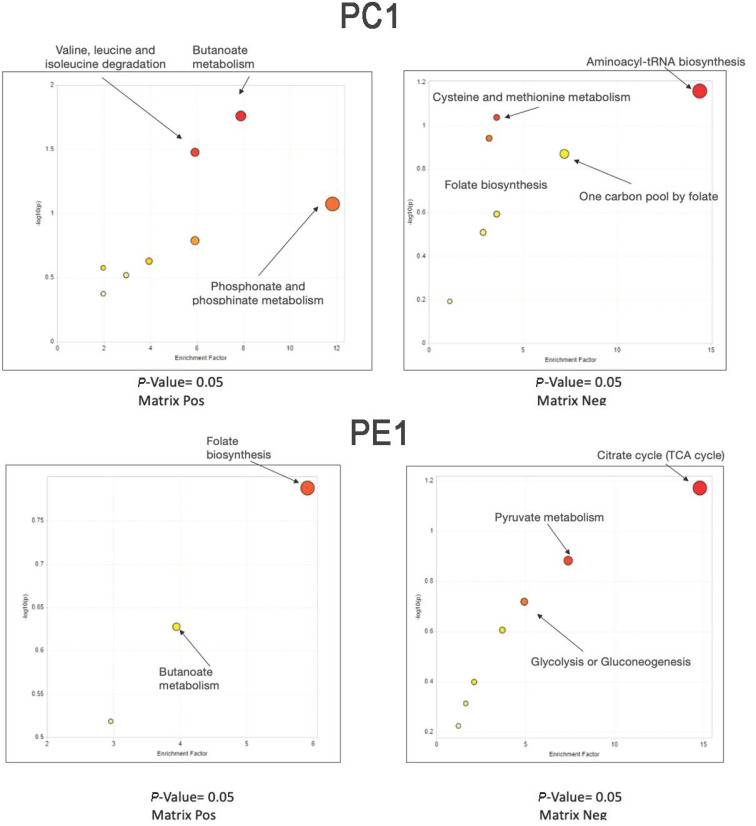

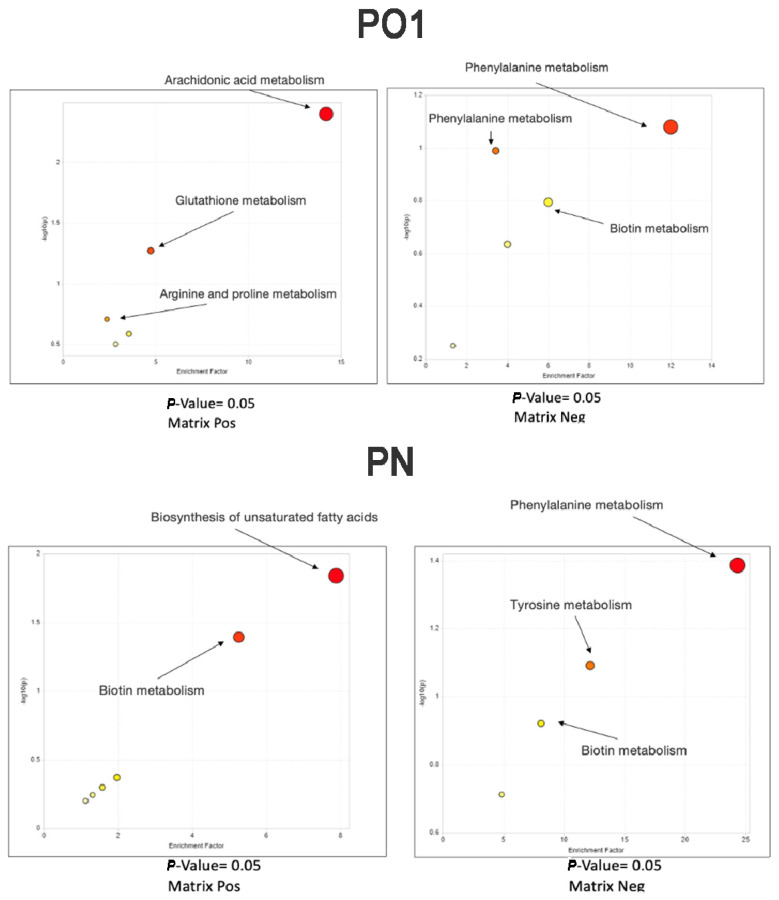

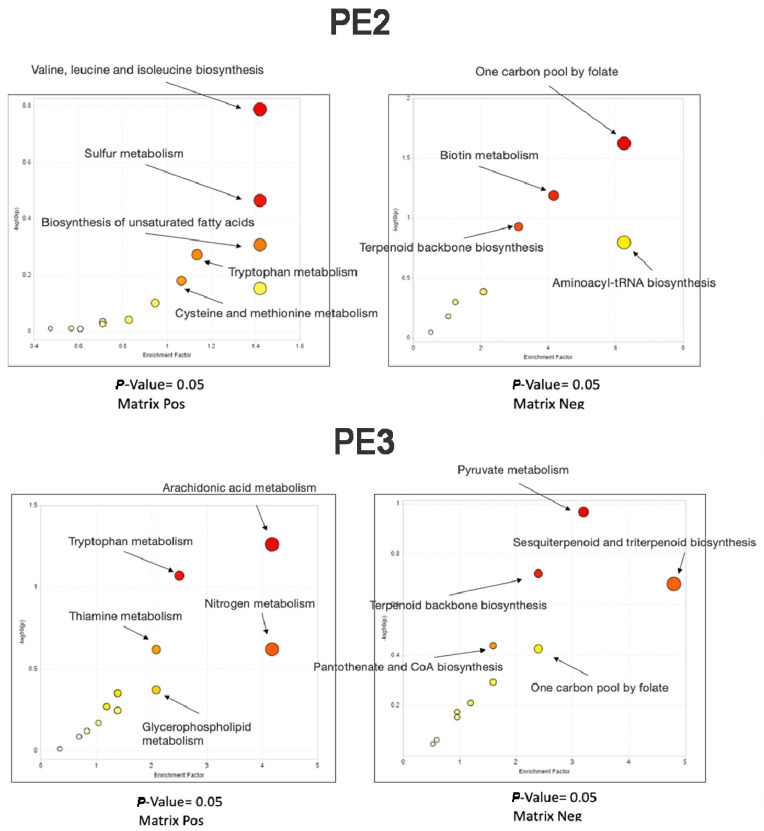

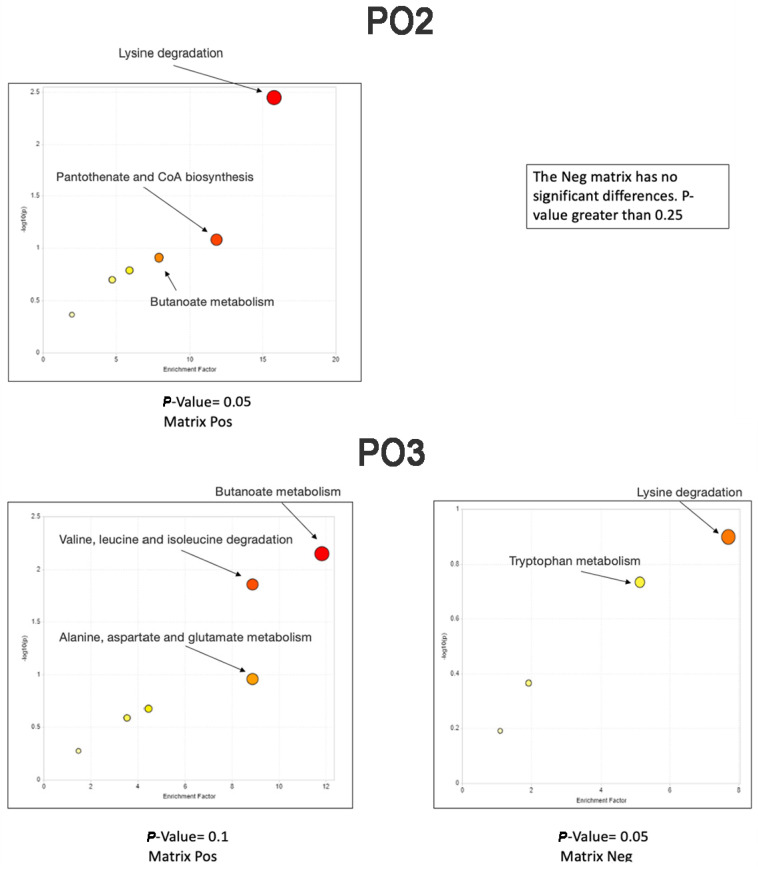

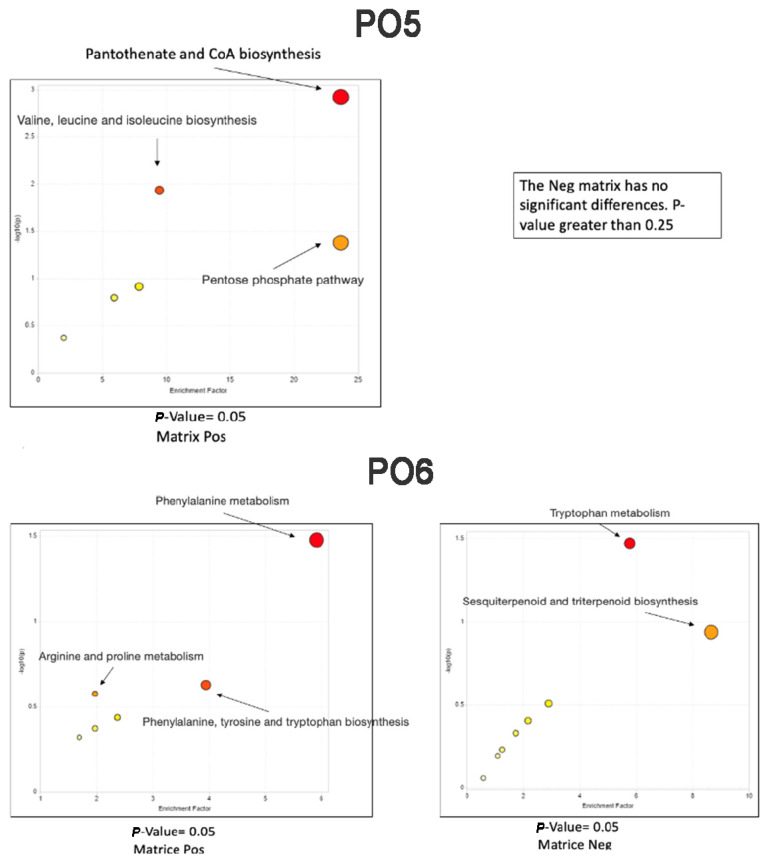
Mummichog Pathway Activity Profile: Comparison of metabolic networks present in samples in both polarities.

A comparative investigation for exploring the effect of different substrates on the metabolic profile was made between *Pleurotus* spp. mycelia were grown in substrate S2 with respect to substrate S1 taken as reference (Figure 1 and Figure 2). The most evident thing was that the metabolic pathways were strongly influenced by the substrate. Some differences can be tentatively explained. For example, folate biosynthesis was greater for sample PE4 grown on substrate S2. Indeed, this substrate contained, among others, wheat straw which had a good content of vitamin B12 and folic acid [39]. Similar evidence was noted for the metabolic pathway of arginine and proline metabolism which was increased in sample PE2 grown on substrate S2. The same substrate was able to activate the arginine and proline pathways, compared to substrate S1.

In these figures, the pathways revealed by the functional analysis are represented by colored circles, whose abscissas correspond to the enrichment factor and ordinates to the -log of the *p*-Value. Also, the size of the circle represents the enrichment factor, the color from yellow to red is proportional to the -log of the p-value. The most regulated pathways are in the upper right-hand corner. One carbon pool by folate pathway was particularly expressed in samples PE2 and PO4, rather in the other *Pleurotus* samples. By contrast, the folate biosynthesis pathway was expressed at higher levels in PE1 and PE4 samples, whereas the pantothenate and CoA biosynthesis pathway were particularly high in the PO5 sample.

### 3.3. Phenolic Composition of The Extracts

The extracts were also investigated through HPLC-DAD-MS to determine the composition of phenolic compounds.

**Figure 3:**
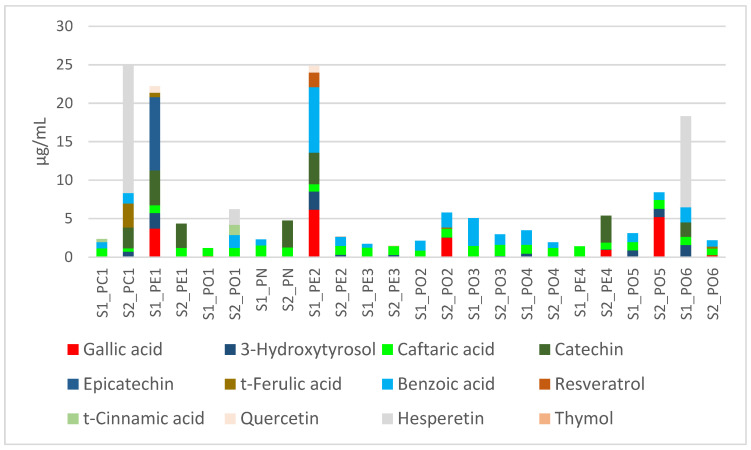
Cumulative distribution of the most abundant phytochemicals in the tested extracts.

Among all tested extracts, 28 compounds have been identified (Supplementary Material: Table S3 and chromatograms). Only caftaric acid was quantified in all extracts (Figure 3). 

Among these phytochemicals, the combination of caftaric acid and benzoic acid was recorded in 9 extracts, while PE1-S2, PN-S2, and PE4-S2 are characterized by the presence of only caftaric acid and catechin. Anyway, there was no effect of the substrate on the qualitative or quantitative composition. 

Regarding *P. ostreatus*, caftaric acid and benzoic acid were the prominent compounds; by contrast in *P. eryngii*, with the only exception of the sample PE3, catechin is the main phytochemical. In *P. nebrodensis* (PN), the presence of caftaric acid was not influenced by the substrate composition, while S2 increased the catechin level. In *P. columbinus* (PC1) cultivated in substrate S2, there was a significant increase in total phenols, as also witnessed by the elevated level of the flavonoid hesperitin. We cannot exclude a possible influence of substrate S2 on the flavonoid biosynthesis pathway (KEGG map 00941), in *P. columbinus.*

As a final remark, the presence of phenolic compounds in these samples was also an index of potential scavenging/reducing and enzyme inhibition properties [40]. Additionally, phenolic compounds have also been demonstrated to exert antimicrobial effects [41,42]. In this context, the phenolic composition of the extracts was consistent with subsequent investigations on antioxidant, enzyme inhibition, and antimicrobial effects.

### 3.4. Antimicrobial Activity

The antimicrobial activity of the extracts is shown in Table 6, Table 7, Table 8, Table 9, Table 10 and Table 11, also in comparison with reference antimicrobial drugs, namely ciprofloxacin, fluconazole, and griseofulvin. All extracts from *Pleurotus* mycelia displayed antimicrobial activity in the concentration range of 1.56 to 200 μg mL^−1^. Regarding the yeasts, *C. parapsilosis* (YEPGA 6551) was the most sensitive strain to the PN-PO6 extracts (S2), with MIC ranges of 7.78–>200 μg mL^−1^, while *C. albicans* (YEPGA 6183) showed the least sensitivity to the mushroom extracts. The results of the growth inhibition of yeast strains highlighted, albeit partially, the major activity of the extract derived from the S2 growth substrate. With reference to bacteria, the strongest inhibition was observed for the *Pleurotus* extracts PC1 and PO5 (S1) [MIC <2.47–>200 μg mL^−1^ against *E. coli* (ATCC 10536) and *B. cereus* PeryMycA 2]. Collectively, Gram-bacterial strains (*E. coli* PeruMyc 2 and 3, *S. typhi* 7, and *P. aeruginosa* ATCC 15442) were less sensitive to mushroom extracts than that of Gram+ ones, as already observed for *F. torulosa* [43]. All results from the tested extracts showed active inhibition of dermatophytes growth. Regarding *A. curreyi* (CCF 5207), *A. insingulare*, and *T. rubrum* (CCF 4933), they were the most sensitive fungal species to all mushroom extracts, with MIC range between 31.49 and 158.74 μg mL^−1^. Values of MIC < 100 μg mL^−1^ were considered as an index of high antimicrobial activity [44].

The antimicrobial activity of *Pleurotus* samples can be hypothesized, albeit partially, as due to the presence of the so-called CP compounds (derivatives of phosphonate and phosphinate with substitution of alkyl group for hydrogen of phosphorus-hydrogen bonds), monobactams, that are beta-lactam antibiotics (containing a monocyclic beta-lactam nucleus) and sesquiterpenoid and triterpenoid, for which the following metabolic pathways have been highlighted: phosphonate and phosphinate metabolism (PC1 and PE2 samples), monobactam biosynthesis (PE2) and sesquiterpenoid and triterpenoid biosynthesis (PE3, PO4, PE4, and PO6), respectively. The presence of these metabolic pathways has never been indicated in the mycelia of *Pleurotus* spp. Many C-P compounds are known bioactive substances used in medicine (antibiotics) and agriculture (herbicide) such as fosfomycin, FR-33289, rhizocticin, and bialaphos, while monobactams are beta-lactam antibiotics containing a monocyclic beta-lactam nucleus. Sesquiterpenoid and triterpenoid (present in PE3, PO4, PE4, and PO6 samples) are a group of terpenoids consisting of three isoprene units known for their antimicrobial capacity [45].

### 3.5. Antiradical Activity

Regarding the antiradical activity, experimental data were normalized and expressed as EC_50_ values (μg mL^−1^) for each mushroom extract and Trolox, which was used as a reference antioxidant compound. The antiradical properties were investigated through both DPPH and ABTS, which are common assays used for measuring the intrinsic antioxidant properties of extracts. The results of these tests are shown in Table 12. Values for DPPH radical scavenging activity varied between 886 and 4871 (μg mL^−1^), with the higher potency demonstrated by sample PE3 cultivated in substrate S1. Values for ABTS radical scavenging activity varied between 87 and 377 (μg mL^−1^), and the best activity was shown by the samples PE2 in both substrates, with mean values in the range 87–94 μg mL^−1^ referred to the EC_50_; thus, suggesting that the substrates can affect the sample properties in different, that cannot be generalized. For instance, in the DDPH test, samples PC1, PO2, PO3, PO4, and PO5 were not influenced by the change of growth substrate. Whilst in the ABTS test, the substrate always influenced the intrinsic activity, with both stimulating or inhibiting antiradical effects. We cannot exclude that the discrepancies observed between DPPH and ABTS tests can be regarded as the differences between ABTS and DPPH radicals. Indeed, ABTS has been described to be more accurate compared with DPPH, when applied to samples rich in hydrophilic, lipophilic, and highly pigmented antioxidant compounds [46]. 

## 4. Conclusions

With the development of technology, liquid chromatography coupled to a mass-spectrometry approach can be widely applied in metabolomic studies currently, having a wide detected range and high specificity and sensitivity [47]. In the present study, this method was used to analyze the metabolic profiling of *Pleurotus* species mycelia, which showed satisfactory data quality. The literature about the characterization of *Pleurotus* metabolic pathways is unexpectedly poor, even for *P. ostreatus* and a few other species. It is almost missing as concerns *P. columbinus*, since it has been only recently accepted as an independent species. The present findings support further investigations aimed at evaluating the influence of growth substrate on *Pleurotus* spp. antimicrobial and antioxidant properties. The extracts from *Pleurotus* revealed valuable sources of primary and secondary metabolites, thus suggesting potential applications in the formulation of food supplements, above all in terms of antioxidant and antimicrobial properties.

Regarding the antimicrobial effects, the results from the present study did not point out the optimal substrate for the cultivation of fungi. However, the effect of the substrate was present and should be deeply considered in view of the production of antioxidant extracts from *Pleurotus* species.

As a concluding remark, in view of a modern concept of sustainability, waste products of agronomical chains can be considered promising substrates for fungal cultivation. Indeed, our results demonstrated that residual plant materials, still containing primary and secondary metabolites, can play pivotal roles in modulating selectively fungal metabolome, with a concomitant influence on the potential use as food and/or health-promoting agent.

Future studies still prove necessary to better define the interactions between plant phytocomplex and fungal response, to drive the cultivation of fungi towards both sustainable improvements of the chain production and search for innovative market products.

## Figures and Tables

**Table 1:** Sample ID and data relating to the location and date of collection of *Pleurotus* species studied.

Sample ID	Species	Locality	Date
PC1	*Pleurotus columbinus* Quél.	Cascata delle Marmore (TR)	Apr 2019
PE1	*Pleurotus eryngii* var. *thapsiae* Venturella, Zervakis & Saitta	Madonie (Piano Zucchi) Palermo	Oct 2019
PO1	*Pleurotus ostreatus* (Jacq.) P. Kumm.	Cima di Tuoro (PG)	Sep 2017
PN	*Pleurotus nebrodensis* (Inzenga) Quél.	Monte Malletto (Etna, CT)	May 2021
PE2	*Pleurotus eryngii* (DC.) Quél.	Etna Sciare S. Venera Maletto	Nov 2021
PE3	*Pleurotus eryngii* var. *ferulae* (Lanzi) Sacc.	Isola Polvese (PG)	Jan 2017
PO2	*Pleurotus ostreatus* (Jacq.) P. Kumm.	Castel Porziano (RM)	Nov 2018
PO3	*Pleurotus ostreatus* (Jacq.) P. Kumm.	Castel Porziano (RM)	Nov 2018
PO4	*Pleurotus ostreatus* (Jacq.) P. Kumm.	Rivotorto (PG)	Oct 2020
PE4	*Pleurotus eryngii* var. *elaeoselini* Venturella, Zervakis & La Rocca	Vallone dei Sieli, Motta sant’Anastasia (Catania)	Nov 2017
PO5	*Pleurotus ostreatus* (Jacq.) P. Kumm.	Monte Peglia (S. Venanzo, TR)	Nov 2017
PO6	*Pleurotus ostreatus* (Jacq.) P. Kumm.	Monte Subasio (PG)	May 2017

**Table 2:** GenBank sequences and identity percentages with different samples of *Pleurotus* species studied.

Species	Sample ID	Base Pair	Correspondence with Genbank Seq.	% Identity	Accession no.
*Pleurotus columbinus*	PC1	621	*Pleurotus columbinus*	100	MG282482.1
*Pleurotus eryngii var. thapsiae*	PE1	345	*Pleurotus eryngii*	99.42	MH517527.1
*Pleurotus ostreatus*	PO1	568	*Pleurotus ostreatus*	100	MT644908.1
*Pleurotus nebrodensis*	PN	616	*Pleurotus nebrodensis*	99.51	KF743821.1
*Pleurotus eryngii var. ferulae*	PE3	511	*Pleurotus eryngii var. ferulae*	99.42	AB286153.1
*Pleurotus ostreatus*	PO2	641	*Pleurotus pulmonarius*	100	MN239983.1
*Pleurotus ostreatus*	PO3	670	*Pleurotus ostreatus*	100	GU186818.1
*Pleurotus eryngii var. elaeoselini*	PE4	636	*Pleurotus eryngii*	100	OPE241308.1
*Pleurotus ostreatus*	PO5	613	*Pleurotus ostreatus*	99.19	MT644908.1
*Pleurotus ostreatus*	PO6	612	*Pleurotus pulmonarius*	99.19	MH810334.1

**Table 3:** Gradient elution conditions of the HPLC-DAD analyses for the identification and quantification of polyphenolic compounds.

Time (Min.)	Composition A% (Water+Formic Acid 0.1%)	Flow (mL/min)
1	97	0.6
5	77	0.6
12	73	0.6
18	57	0.6
25	52	0.6
32	50	0.6
34	50	0.6
37	35	0.6
40	5	0.6
47	5	0.6
48	97	0.6
60	97	0.6

**Table 4:** Phenolic compounds analyzed.

	Peak Name	Retention Time
1	Gallic acid	8.80
2	3-Hydroxytyrosol	11.71
3	Caftaric acid	12.93
4	Catechin	14.80
5	4-Hydroxybenzoic acid	16.20
6	Loganic acid	16.60
7	Chlorogenic acid	16.81
8	Vanillic acid	18.60
9	Caffeic acid	19.00
10	Epicatechin	19.41
11	Syringic acid	20.05
12	p-Coumaric acid	23.06
13	t-Ferulic acid	24.00
14	Benzoic acid	26.38
15	Hyperoside	26.92
16	Rutin	27.16
17	Isoquercetin	27.29
18	Resveratrol	27.70
19	Rosmarinic acid	28.53
20	t-Cinnamic acid	34.39
21	Quercetin	35.89
22	Hesperetin	39.38
23	Kaempferol	41.74
24	Carvacrol	44.69
25	Thymol	44.92
26	Flavone	45.60
27	3-Hydroxyflavone	46.05
28	Emodin	47.70

**Table 5:** Regulated (+) or unregulated (-) metabolic pathway between *Pleurotus* samples as an effect of the different growth substrates.

ID Sample	PC1	PE1	PO1	PN	PE2	PE3	PO2	PO3	PO4	PE4	PO5	PO6	KEGG Pathway Map
Metabolic Pathway *													
Aminoacid metabolism													
Valine, leucine, and isoleucine biosynthesis	+	-	-	-	+	+	+	-	-	+	+	-	map00290
Glycine, serine, and threonine metabolism	+	-	-	-	+	-	-	-	+	+	-	-	map00260
Tyrosine metabolism	-	-	+	+	-	-	-	-	-	-	-	-	map00350
Tryptophan metabolism	-	-	-	+	+	+	-	+	-	+	-	+	map00380
Arginine biosynthesis	+	+	+	+	+	+	-	+	-	+	-	+	map00220
Valine, leucine and isoleucine degradation	-	-	-	-	+	+	+	+	-	+	+	-	map00280
Lysine biosynthesis	-	-	-	-	+	-	+	+	-	+	+	-	map00300
Phenylalanine metabolism	-	-	+	+	+	+	-	-	-	+	-	+	map00360
Thiamine metabolism	-	+	-	+	+	+	-	+	-	+	-	-	map00730
Histidine metabolism	-	-	-	+	+	+	-	-	+	+	-	+	map00340
Cysteine and methionine metabolism	+	+	-	-	+	+	-	+	-	+	-	+	map00270
Phenylalanine, tyrosine, and tryptophan biosynthesis	-	+	-	-	+	+	-	-	-	+	-	+	map00400
Arginine and proline metabolism	+	+	+	-	+	+	+	+	+	+	+	+	map00330
Glutathione metabolism	+	-	+	-	+	+	-	-	+	+	-	-	map00480
beta-Alanine metabolism	-	-	+	-	+	+	-	-	+	-	-	-	map00410
Lysine degradation	-	-	-	-	+	-	+	+	-	+	-	-	map00310
Alanine, aspartate and glutamate metabolism	+	-	-	-	-	+	-	+	+	+	-	+	map00250
Amino sugar and nucleotide sugar metabolism													
Amino sugar and nucleotide sugar metabolism	-	+	-	-	+	-	-	-	-	+	-	-	map00520
Glycolysis or Gluconeogenesis	-	+	-	-	+	+	-	-	-	-	-	-	map00010
Pentose phosphate pathway	-	-	+	-	+	-	-	-	-	+	+	-	map00030
Fructose and mannose metabolism	-	-	-	-	+	+	-	-	-	-	-	-	map00051
Galactose metabolism	-	-	-	-	+	-	-	-	-	+	-	-	map00052
Pentose and glucuronate interconversions	-	-	-	-	+	-	-	-	-	-	-	-	map00040
N-Glycan biosynthesis	-	-	-	-	+	-	-	-	-	-	-	-	map00510
Starch and sucrose metabolism	-	-	-	-	+	-	-	-	-	+	-	-	map00500
Nucleotide metabolism													
Purine metabolism	+	+	-	-	+	+	-	-	+	+	-	+	map00230
Pyrimidine metabolism	+	-	-	+	+	+	-	+	+	+	-	+	map00240
Aminoacyl-tRNA biosynthesis	+	-	-	+	+	+	-	-	+	+	-	+	map00970
Lipid metabolism													
Inositol phosphate metabolism	-	-	-	-	+	-	-	-	-		-	-	map00562
Phosphatidylinositol signaling system	-	-	-	-	+	-	-	-	-	-	-	-	map04070
Glycerophospholipid metabolism	-	-	-	-	+	+	-	-	-	+	-	-	map00564
Sphingolipid metabolism	-	-	-	-	+	+	-	-	-	+	-	-	map00600
Vitamin metabolism													
Nicotinate and nicotinamide metabolism	-	-	+	-	+	-	-	-	+	+	-	-	map00760
Cyanoamino acid metabolism	-	-	-	-	-	-	-	-	-	+	-	-	map00460
Riboflavin metabolism	-	-	-	-	-	-	-	-	-	+	-	-	map00740
Vitamin B6 metabolism	-	-	-	-	+	-	-	-	-	+	-	+	map00750
Pantothenate and CoA biosynthesis	-	-	+	-	+	+	+	-	-	+	+	+	map00770
Folate biosynthesis	+	+	-	-	+	+	-	-	+	+	-	-	map00790
One carbon pool by folate	+	-	+	-	+	+	-	-	+	+	-	-	map00670
Fatty acid metabolism													
Biosynthesis of unsaturated fatty acids	-	-	-	+	+	-	-	-	-	+	-	-	map01040
Arachidonic acid metabolism	-	-	+	-	+	+	-	-	-	+	-	-	map00590
Steroid biosynthesis	-	-	-	-	+	+	-	-	+	+	-	+	map00905
Terpenoid metabolism													
Terpenoid backbone biosynthesis	-	+	-	-	+	+	-	-	+	+	-	+	map00909
Sesquiterpenoid and triterpenoid biosynthesis	-	-	-	-	-	+	-	-	+	+	-	+	map00909
Other metabolic pathways													
Porphyrin and chlorophyll metabolism	-	-	-	-	+	-	-	-	-	+	-	-	map00860
Pyruvate metabolism	-	+	+	-	+	+	-	-	-	+	-	-	map00620
Citrate cycle (TCA cycle)	-	+	-	-	-	+	-				-	-	map00020
Glyoxylate and dicarboxylate metabolism	-	-	-	-	-	+	-	-	+	-	-	-	map00630
Butanoate metabolism	-	+	-	-	+	-	+	+	+	+	-	-	map00650
Phosphonate and phosphinate metabolism	+	-	-	-	+	-	-	-	-	-	-	-	map00440
Monobactam biosynthesis	-	-	-	-	+	-	-	-	-	-	-	-	map00261
Methane metabolism	+	-	-	-	+	-	-	-	+	+	-	-	map00680
Nitrogen metabolism	-	-	-	-	-	+	-	-	+	-	-	-	map00910

* metabolic pathway absent (-), metabolic pathway present (+).

**Table 6:** Minimal inhibitory concentrations (MICs) of *Pleurotus* mycelia (S1) extracts against bacteria isolates.

	MIC (µg mL^−1^) *							
	*Escherichia* *coli*	*Escherichia* *coli*	*Escherichia* *coli*	*Bacillus* *cereus*	*Pseudomonas* *aeruginosa*	*Bacillus* *subtilis*	*Salmonella* *typhi*	*Staphylococcus* *aureus*
Bacteria	(ATCC 10536)	(PeruMycA 2)	(PeruMycA 3)	(PeruMycA 4)	(ATCC 15442)	(PeruMycA 6)	(PeruMycA 7)	(ATCC 6538)
PC1	125.99 (100–200)	2.47 (1.56–3.12)	>200	7.87 (6.26–12.5)	>200	>200	>200	>200
PE1	9.92 (6.25–12.5)	3.93 (3.12–6.25)	>200	4.92 (3.125–6.25)	3.93 (3.12–6.25)	4.96 (3.125–6.25)	>200	19.84 (12.5–25)
PO1	79.37 (50–100)	79.37 (50–100)	158.74	>200	>200	>200	>200	>200
PN	2.47 (1.56–3.12)	3.93 (3.12–6.25)	>200	7.87 (6.25–12.5)	7.87 (6.25–12.5)	3.93 (3.12–6.25)	>200	7.87 (6.25–12.5)
PE2	3.93 (3.12–6.25)	3.93 (3.12–6.25)	>200	3.93 (3.12–6.25)	7.87 (6.25–12.5)	3.93 (3.12–6.25)	>200	16.74 (12.5–25)
PE3	9.92 (6.25–12.5)	7.87 (6.25–12.5)	>200	2.47 (1.56–3.12)	3.93 (3.12–6.25)	7.87 (6.25–12.5)	>200	9.92 (6.25–12.5)
PO2	39.68 (25–50)	79.37 (50–100)	>200	158.74 (100–200)	158.74 (100–200)	>200	>200	>200
PO3	158.74 (100–200)	3.93 (3.12–6.25)	>200	79.37 (50–100)	>200	>200	>200	>200
PO4	39.68 (25–50)	7.87 (6.25–12.5)	>200	15.74 (12.5–25)	>200	>200	>200	>200
PE4	2.47 (1.56–3.12)	3.93 (3.12–6.25)	>200	3.93 (3.12–6.25)	7.87 (6.25–12.5)	<6.25	>200	9.92 (6.25–12.5)
PO5	3.93 (3.12–6.25)	>200	>200	125.99 (100–200)	>200	>200	>200	>200
PO6	1.96 (1.56–3.12)	31.49 (25–50)	>200	125.99 (100–200)	>200	>200	>200	>200
Ciprofloxacin (µg mL^−1^ )	31.49 (25–50)	9.92 (6.25–12.5)	79.37 (50–100)	125.99 (100–200)	125.99 (100–200)	125.99 (100–200)	79.37 (50–100)	200- > 200

* Mic values are reported as geometric means of three independent replicates (n = 3). MIC range concentrations are reported within brackets.

**Table 7:** Minimal inhibitory concentrations (MICs) of *Pleurotus* mycelia (S2) extracts against bacteria isolates.

	MIC (µg mL^−1^) *							
	*Escherichia* *coli*	*Escherichia* *coli*	*Escherichia* *coli*	*Bacillus* *cereus*	*Pseudomonas* *aeruginosa*	*Bacillus* *subtilis*	*Salmonella* *typhy*	*Staphylococcus* *aureus*
Bacteria	(ATCC 10536)	(PeruMycA 2)	(PeruMycA 3)	(PeruMycA 4)	(ATCC 15442)	(PeruMycA 6)	(PeruMycA 7)	(ATCC 6538)
PC1	79.37 (50–100)	125.99 (100–200)	125.99 (100–200)	125.99 (100–200)	125.99 (100–200)	125.99 (100–200)	79.37 (50–100)	125.99 (100–200)
PE1	3.93 (3.125–6.25)	15.75 (12.5–25)	>200	3.93 (3.125–6.25)	62.99 (50–100)	31.49 (25–50	>200	158.74 (100–200)
PO1	158.74 (100–200)	79.37 (100–200)	158.74 (100–200)	158.74 (100–200)	125.99 (100–200)	158.74 (100–200)	39.68 (25–50)	158.74 (100–200)
PN	4.96 (3.125–6.25)	9.92 (6.25–12.5)	>200	3.93 (3.125–6.25)	79.37 (50–100)	15.75 (12.5–25)	>200	125.99 (100–200)
PE2	4.96 (3.125–6.25)	7.87 (6.25–12.5)	>200	9.92 (6.25–12.5)	39.68 (25–50)	9.92 (6.25–12.5)	>200	62.99 (50–100)
PE3	7.87 (6.25–12.5)	9.92 (6.25–12.5)	>200	39.68 (25–50)	62.99 (50–100)	15.75 (12.5–25)	>200	158.74 (100–200)
PO2	125.99 (100–200)	158.74 (100–200)	79.37 (50–100)	62.99 (50–100)	125.99 (100–200)	158.74 (100–200)	125.99 (100–200)	62.99 (50–100)
PO3	125.99 (100–200)	125.99 (100–200)	79.37 (50–100)	125.99 (100–200)	158.74 (100–200)	158.74 (100–200)	125.99 (100–200)	79.37 (50–100)
PO4	158.74 (100–200)	158.74 (100–200)	125.99 (100–200)	125.99 (100–200)	79.37 (50–100)	>200	79.37 (50–100)	62.99 (50–100)
PE4	3.93 (3.125–6.25)	3.93 (3.125–6.25)	>200	15.75 (12.5–25)	62.99 (50–100)	19.84 (12.5–25)	>200	62.99 (50–100)
PO5	158.74 (100–200)	>200	>200	158.74 (100–200)	>200	>200	>200	>200
PO6	79.37 (50–100)	62.99 (50–100)	>200	158.74 (100–200)	>200	>200	>200	>200
Ciprofloxacin (µg mL^−1^)	31.49 (25–50)	9.92 (6.25–12.5)	79.37 (50–100)	125.99 (100–200)	125.99 (100–200)	125.99 (100–200)	79.37 (50–100)	200- > 200

* Mic values are reported as geometric means of three independent replicates (n = 3). MIC range concentrations are reported within brackets.

**Table 8:** Minimal inhibitory concentrations of *Pleurotus* mycelia (S1) extracts against yeast isolates.

	MIC (µg mL^−1^) *			
	*Candida tropicalis*	*Candida albicans*	*Candida parapsilosis*	*Candida albicans*
Yeast Strain	(YEPGA 6184)	(YEPGA 6379)	(YEPGA 6551)	(YEPGA 6183)
PC1	158.74 (100–200)	158.74 (100–200)	158.74 (100–200)	>200
PE1	>200	158.74 (100–200)	15.74 (12.5–25)	>200
PO1	>200	158.74 (100–200)	158.74 (100–200)	158.74 (100–200)
PN	>200	>200	7.87 (6.25–12.5)	>200
PE2	>200	>200	9.92 (6.25–12.5)	>200
PE3	>200	>200	7.87 (6.25–12.5)	>200
PO2	158.74 (100–200)	>200	125.99 (100–200)	>200
PO3	158.74 (100–200)	>200	>200	158.74 (100–200)
PO4	158.74 (100–200)	>200	>200	>200
PE4	>200	>200	7.87 (6.25–12.5)	>200
PO5	158.74 (100–200)	>200	158.74 (100–200)	>200
PO6	>200	158.74 (100–200)	158.74 (100–200)	158.74 (100–200)
Fluconazole (µg mL^−1^)	2	1	4	2

* Mic values are reported as geometric means of three independent replicates (n = 3). MIC range concentrations are reported within brackets.

**Table 9:** Minimal inhibitory concentrations of *Pleurotus* mycelia (S2) extracts against yeast isolates.

	MIC (µg mL^−1^) *			
	*Candida tropicalis*	*Candida albicans*	*Candida parapsilosis*	*Candida albicans*
Yeast Strain	(YEPGA 6184)	(YEPGA 6379)	(YEPGA 6551)	(YEPGA 6183)
PC1	79.37	>200	39.68 (25–50)	>200
PE1	158.74 (100–200)	158.74 (100–200)	15.74	>200
PO1	>200	>200	62.99 (50–100)	>200
PN	>200	>200	7.87 (6.25–12.5)	>200
PE2	>200	>200	7.87 (6.25–12.5)	>200
PE3	>200	>200	7.87 (6.25–12.5)	>200
PO2	158.74 (100–200)	158.74 (100–200)	158.74 (100–200)	>200
PO3	158.74 (100–200)	>200	>200	62.99 (50–100)
PO4	158.74 (100–200)	>200	79.37 (50–100)	>200
PE4	>200	>200	9.92 (6.25–12.5)	>200
PO5	158.74 (100–200)	158.74 (100–200)	125.99 (100–200)	>200
PO6	158.74 (100–200)	158.74 (100–200)	>200	158.74 (100–200)
Fluconazole (µg mL^−1^)	2	1	4	2

* Mic values are reported as geometric means of three independent replicates (n = 3). MIC range concentrations are reported within brackets.

**Table 10:** Minimal inhibitory concentrations (MICs) of *Pleurotus* mycelia (S1) extracts against dermatophyte isolates.

	MIC (µg mL^−1^) *							
	*Trichophyton* *mentagrophytes*	*Trichophyton* *tonsurans*	*Trichophyton* *rubrum*	*Arthroderma* *quadrifidum*	*Trichophyton* *mentagrophytes*	*Arthroderma* *gypseum*	*Arthroderma* *curreyi*	*Arthroderma* *insingulare*
Dermatophyte	(CCF 4823)	(CCF 4834)	(CCF 4933)	(CCF 5792)	(CCF 5930)	(CCF 6261)	(CCF 5207)	(CCF 5417)
PC1	>200	>200	158.74 (100–200)	125.99 (100–200)	125.99 (100–200)	125.99 (100–200)	62.99 (50–100)	79.37 (50–100)
PE1	>200	>200	158.74 (100–200)	79.37 (50–100)	158.74 (100–200)	>200	79.37 (50–100)	125.99 (100–200)
PO1	>200	158.74 (100–200)	125.99 (100–200)	125.99 (100–200)	125.99 (100–200)	>200	62.99 (50–100)	79.37 (50–100)
PN	125.99 (100–200)	158.74 (100–200)	125.99 (100–200)	79.37 (50–100)	158.74 (100–200)	>200	>200	>200
PE2	125.99 (100–200)	>200	158.74 (100–200)	>200	>200	125.99 (100–200)	79.37 (50–100)	79.37 (50–100)
PE3	79.37 (50–100)	158.74 (100–200)	125.99 (100–200)	125.99 (100–200)	158.74 (100–200)	158.74 (100–200)	79.37 (50–100)	79.37 (50–100)
PO2	79.37 (50–100)	>200	125.99 (100–200)	79.37 (50–100)	158.74 (100–200)	>200	31.49 (25–50)	62.99 (50–100)
PO3	158.74 (100–200)	158.74 (100–200)	>200	125.99 (100–200)	>200	>200	79.37 (50–100)	125.99 (100–200)
PO4	>200	>200	158.74 (100–200)	125.99 (100–200)	158.74 (100–200)	>200	79.37 (50–100)	125.99 (100–200)
PE4	158.74 (100–200)	125.99 (100–200)	79.37 (50–100)	>200	125.99 (100–200)	158.74 (100–200)	125.99 (100–200)	79.37 (50–100)
PO5	>200	>200	>200	>200	>200	>200	62.99 (50–100)	79.37 (50–100)
PO6	79.37 (50–100)	>200	79.37 (50–100)	125.99 (100–200)	79.37 (50–100)	158.74 (100–200)	31.49 (25–50)	39.68 (25–50)
Griseofulvin µg mL^−1^	2.52 (2–4)	0.198 (0.125–0.25)	1.26 (1–2)	>8	3.174 (2–4)	1.587 (1–2)	>8	>8

* Mic values are reported as geometric means of three independent replicates (n = 3). MIC range concentrations are reported within brackets.

**Table 11:** Minimal inhibitory concentrations (MICs) of *Pleurotus* mycelia (S2) extracts against dermatophyte isolates.

	MIC (µg mL^−1^) *							
	*Trichophyton* *mentagrophytes*	*Trichophyton* *tonsurans*	*Trichophyton* *rubrum*	*Arthroderma* *quadrifidum*	*Trichophyton* *mentagrophytes*	*Arthroderma* *gypseum*	*Arthroderma* *curreyi*	*Arthroderma* *insingulare*
Dermatophyte	(CCF 4823)	(CCF 4834)	(CCF 4933)	(CCF 5792)	(CCF 5930)	(CCF 6261)	(CCF 5207)	(CCF 5417)
PC1	125.99 (100–200)	158.74 (100–200)	158.74 (100–200)	158.74 (100–200)	>200	>200	31.49 (25–50)	>200
PE1	62.99 (50–10)	158.74 (100–200)	39.68 (25–50)	39.68 (25–50)	>200	62.99 (50–100)	39.68 (25–50)	62.99 (50–100)
PO1	>200	125.99 (100–200)	158.74 (100–200)	>200	125.99 (100–200)	158.74 (100–200)	39.68 (25–50)	125.99 (100–200)
PN	125.99 (100–200)	79.37 (50–100)	31.49 (25–50)	>200	158.74 (100–200)	158.74 (100–200)	62.99 (50–100)	>200
PE2	158.74 (100–200)	125.99 (100–200)	79.37 (50–100)	125.99 (100–200)	>200	125.99 (100–200)	62.99 (50–100)	79.37 (50–100)
PE3	>200	158.74 (100–200)	125.99 (100–200)	158.74 (100–200)	>200	158.74 (100–200)	125.99 (100–200)	125.99 (100–200)
PO2	158.74 (100–200)	>200	125.99 (100–200)	125.99 (100–200)	158.74 (100–200)	>200	158.74 (100–200)	79.37 (50–100)
PO3	>200	>200	79.37 (50–100)	125.99 (100–200)	>200	>200	79.37 (50–100)	79.37 (50–100)
PO4	158.74 (100–200)	158.74 (100–200)	125.99 (100–200)	125.99 (100–200)	158.74 (100–200)	>200	79.37 (50–100)	62.99 (50–100)
PE4	>200	>200	79.37 (50–100)	>200	125.99 (100–200)	79.37 (50–100)	62.99 (50–100)	125.99 (100–200)
PO5	>200	158.74 (100–200)	158.74 (100–200)	158.74 (100–200)	>200	>200	62.99 (50–100)	158.74 (100–200)
PO6	>200	>200	125.99 (100–200)	125.99 (100–200)	>200	>200	125.99 (100–200)	79.37 (50–100)
Griseofulvin (µg mL^−1^)	2.52 (2–4)	0.198 (0.125–0.25)	1.26 (1–2)	>8	3.174 (2–4)	1.587 (1–2)	>8	>8

* Mic values are reported as geometric means of three independent replicates (n = 3). MIC range concentrations are reported within brackets.

**Table 12:** Antiradical properties of the tested *Pleurotus* extracts.

	DPPH Test		ABTS Test	
Sample	EC50		EC50	
	µg mL^−1^	Trolox eq.	µg mL^−1^	Trolox eq.
PC1-S1	3248 ± 388	650 ± 78	152 ± 18	38 ± 5
PE1-S1	4871 ± 538	974 ± 108	103 ± 12	26 ± 3
PO1-S1	4871 ± 525	974 ± 105	133 ± 16	33 ± 4
PN-S1	1392 ± 168	278 ± 34	97 ± 12	24 ± 3
PE2-S1	2436 ± 294	487 ± 59	87 ± 10	22 ± 3
PE3-S1	886 ± 103	177 ± 21	135 ± 16	34 ± 4
PO2-S1	3248 ± 353	650 ± 71	189 ± 22	47 ± 6
PO3-S1	3248 ± 368	650 ± 74	374 ± 41	87 ± 10
PO4-S1	3248 ± 360	650 ± 72	170 ± 20	43 ± 5
PE4-S1	1949 ± 235	390 ± 47	271 ± 32	68 ± 8
PO5-S1	2436 ± 292	487 ± 58	101 ± 12	25 ± 3
PO6-S1	1949 ± 277	390 ± 46	131 ± 16	33 ± 4
PC1-S2	3248 ± 375	650 ± 75	234 ± 28	59 ± 7
PE1-S2	1949 ± 218	390 ± 44	189 ± 22	47 ± 6
PO1-S2	2436 ± 279	487 ± 56	100 ± 12	25 ± 3
PN-S2	1392 ± 152	278 ± 30	167 ± 20	42 ± 5
PE2-S2	1392 ± 154	278 ± 31	94 ± 11	24 ± 3
PE3-S2	1624 ± 178	354 ± 36	91 ± 11	23 ± 3
PO2-S2	3248 ± 390	650 ± 78	147 ± 17	37 ± 4
PO3-S2	3248 ± 397	650 ± 79	255 ± 30	64 ± 8
PO4-S2	3248 ± 391	650 ± 78	377 ± 45	94 ± 11
PE4-S2	2436 ± 289	487 ± 58	263 ± 31	66 ± 8
PO5-S2	2436 ± 287	487 ± 57	119 ± 14	30 ± 4
PO6-S2	3248 ± 386	650 ± 77	206 ± 22	52 ± 6

## Data Availability

Original data are available from the corresponding author.

## Supplementary Materials

The following supporting information can be downloaded at: https://www.mdpi.com/article/10.3390/antibiotics11111468/s1. Preparation of *Pleurotus* samples: Tables S1 and S2. Quantitative analysis of phenolic compounds: Table S3 and chromatograms.
