# Supplementary material for: Untargeted Metabolomics Used to Describe the Chemical Composition, Antioxidant and Antimicrobial Effects of Extracts from Pleurotus spp. Mycelium Grown in Different Culture Media
Source: Antibiotics (Basel). 2022 Oct 25;11(11):1468. doi: 10.3390/antibiotics11111468 (PMC9686522; doi:10.3390/antibiotics11111468)
Supplement: Supplementary file 1 [file antibiotics-11-01468-s001.zip › S1_PC1.pdf]

Chromatogram

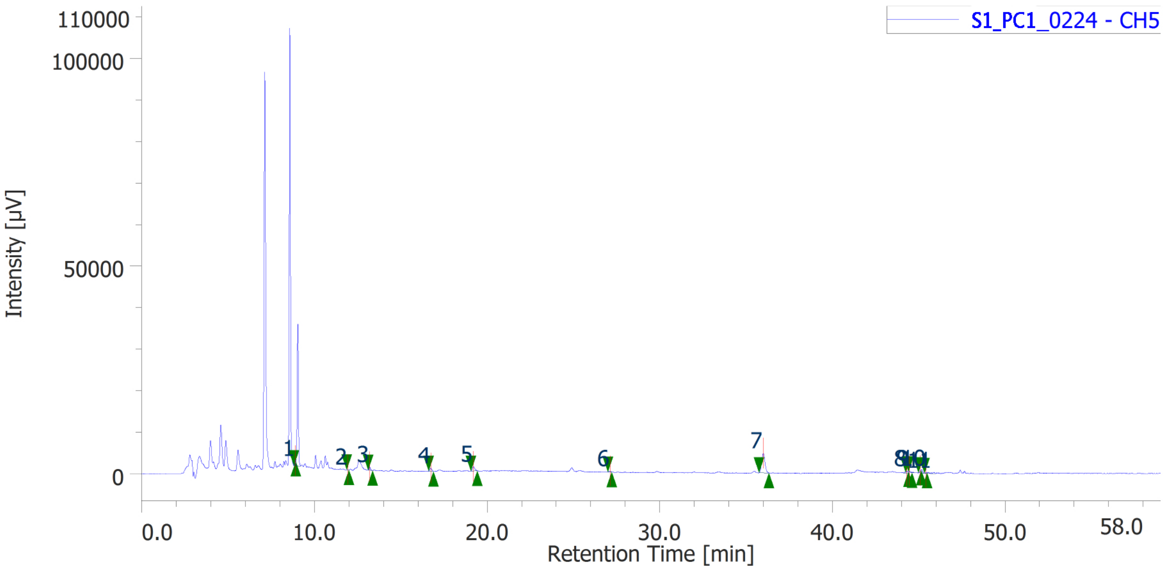

Chromatogram Information	
User Name	Administrator
Date Modified	3/14/2022 11:18:47 AM
Description	
HPLC System Name	HPLC2_DAD
Injection Date	2/25/2022 4:24:24 PM
Volume	5.00 [µL]
Sample #	12
Project Name	Phenols_08_03_2022
Acquisition Time	59.0 [min]
Acquisition Sequence	
Control Method	
Peak ID Table	PeakID_PhenolicCompound_18_02_2022_86
Calibration Method	Phenols_QUANT_CALIB_05_03_2022_Ch5-9
Additional Information	

Channel & Peak Information Table

Chromatogram Name	S1-PC1_0224-CH5
Sample Name	
Channel Name	254.0nm
Sampling Interval	200 [msec]
Peak Method	PeakMethod_05_03_2022_Extracts (Manual)

#	Peak Name	CH	tR [min]	Area [µV·sec]	Height [µV]	Quantity
1	Gallic acid	5	8.897	4407	1217	N/A
2	3-Hydroxytyrosol	5	11.933	239	72	N/A
3	Caftaric acid	5	13.197	4609	811	1.13593 [ug/mL]
4	4-Hydroxybenzoic acid	5	16.737	5670	701	N/A
5	Vanillic acid	5	19.207	10106	1025	N/A
6	Benzoic acid	5	27.117	1752	212	0.810720 [ug/mL]
7	t-Cinnamic acid	5	35.983	62047	4682	0.408235 [ug/mL]
8	Carvacrol	5	44.293	3384	476	N/A
9	Thymol	5	44.447	3766	481	N/A
10	Flavone	5	45.077	3570	559	N/A
11	3-Hydroxyflavone	5	45.407	978	213	N/A
