# Supplementary material for: Untargeted Metabolomics Used to Describe the Chemical Composition, Antioxidant and Antimicrobial Effects of Extracts from Pleurotus spp. Mycelium Grown in Different Culture Media
Source: Antibiotics (Basel). 2022 Oct 25;11(11):1468. doi: 10.3390/antibiotics11111468 (PMC9686522; doi:10.3390/antibiotics11111468)
Supplement: Supplementary file 1 [file antibiotics-11-01468-s001.zip › S1_PE1.pdf]

Chromatogram

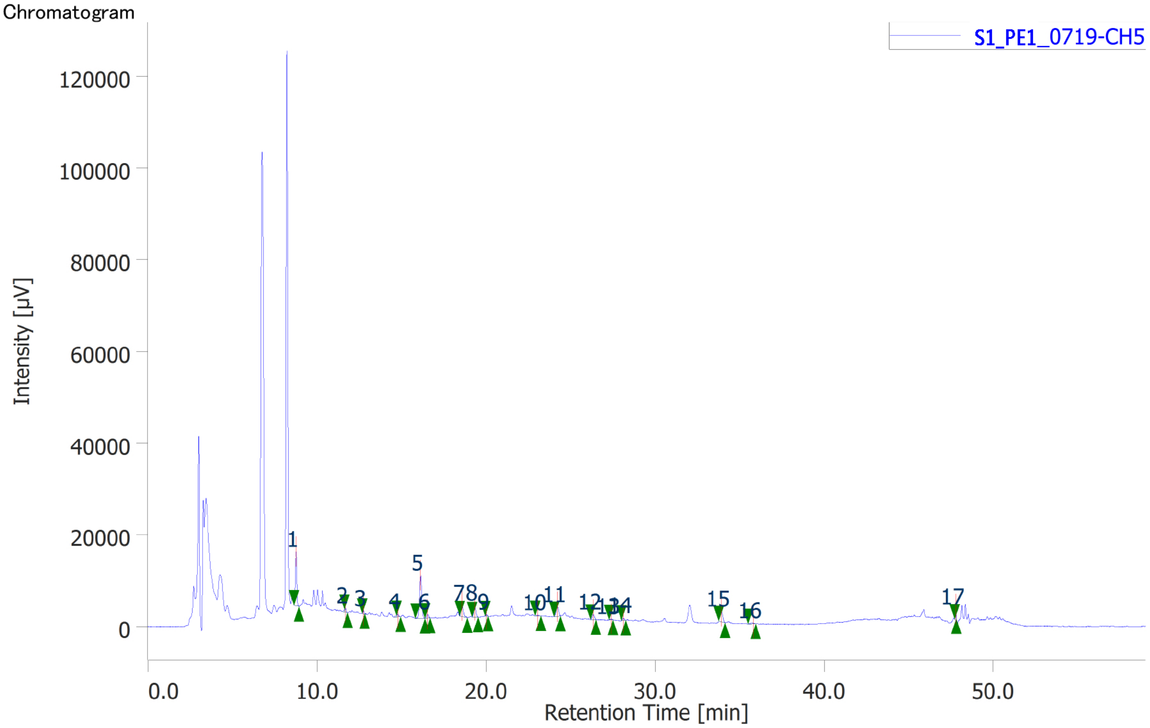

Peak Information

#	Peak Name	CH	tR [min]	Area [μV·sec]	Height [μV]	Area%	Height%	Quantity	Resolution
1	Gallic acid	5	8.750	68217	11788	23.454	30.789	3.70209 [ug/mL]	N/A
2	3-Hydroxytyrosol	5	11.680	5261	898	1.809	2.346	2.01849 [ug/mL]	N/A
3	Caftaric acid	5	12.737	3167	628	1.089	1.641	1.01208 [ug/mL]	11.891
4	Catechin	5	14.773	4338	629	1.491	1.643	4.55133 [ug/mL]	6.168
5	4-Hydroxybenzoic acid	5	16.120	83859	9354	28.832	24.430	0.161037 [ug/mL]	1.911
6	Loganic acid	5	16.533	5696	756	1.958	1.975	N/A	9.046
7	Vanillic acid	5	18.583	23117	2382	7.948	6.221	N/A	3.164
8	Epicatechin	5	19.323	20434	2494	7.026	6.514	9.48965 [ug/mL]	3.628
9	Syringic acid	5	20.013	2160	369	0.743	0.963	N/A	15.095
10	p-Coumaric acid	5	23.043	1472	158	0.506	0.412	N/A	4.568
11	t-Ferulic acid	5	24.220	20923	2029	7.194	5.300	0.584923 [ug/mL]	8.073
12	Benzoic acid	5	26.323	9852	1095	3.387	2.860	3.77952 [ug/mL]	4.622
13	Isoquercetin	5	27.400	1608	204	0.553	0.534	N/A	3.206
14	Resveratrol	5	28.110	3887	507	1.336	1.325	0.564954 [ug/mL]	21.921
15	t-Cinnamic acid	5	33.900	25453	2268	8.751	5.924	N/A	4.657
16	Quercetin	5	35.783	2991	184	1.028	0.480	0.833611 [ug/mL]	40.363
17	Emodin	5	47.767	8419	2543	2.895	6.642	N/A	N/A
