# Supplementary material for: Untargeted Metabolomics Used to Describe the Chemical Composition, Antioxidant and Antimicrobial Effects of Extracts from Pleurotus spp. Mycelium Grown in Different Culture Media
Source: Antibiotics (Basel). 2022 Oct 25;11(11):1468. doi: 10.3390/antibiotics11111468 (PMC9686522; doi:10.3390/antibiotics11111468)
Supplement: Supplementary file 1 [file antibiotics-11-01468-s001.zip › S1_PE2.pdf]

Chromatogram

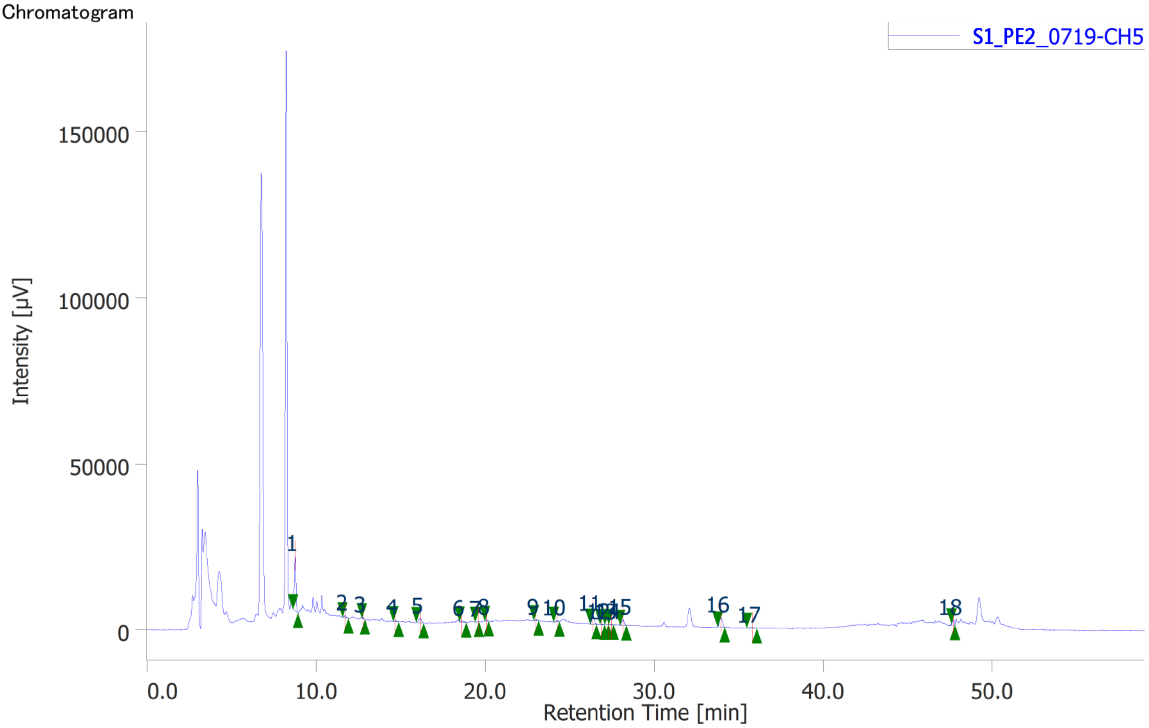

Peak Information

#	Peak Name	CH	tR [min]	Area [µV·sec]	Height [µV]	Area%	Height%	Quantity	Resolution
1	Gallic acid	5	8.753	107234	16507	44.681	54.836	6.17875 [ug/mL]	15.117
2	3-Hydroxytyrosol	5	11.717	5969	660	2.487	2.194	2.35742 [ug/mL]	5.910
3	Caftaric acid	5	12.777	2378	465	0.991	1.544	0.944342 [ug/mL]	10.874
4	Catechin	5	14.687	3875	465	1.614	1.546	4.09636 [ug/mL]	5.773
5	4-Hydroxybenzoic acid	5	16.173	15166	1349	6.319	4.482	N/A	7.248
6	Vanillic acid	5	18.607	4403	361	1.835	1.199	N/A	3.959
7	Epicatechin	5	19.567	376	88	0.157	0.291	N/A	3.719
8	Syringic acid	5	20.103	2504	403	1.043	1.340	N/A	12.884
9	p-Coumaric acid	5	23.007	2595	278	1.081	0.925	N/A	4.633
10	t-Ferulic acid	5	24.247	4016	418	1.673	1.388	N/A	8.483
11	Benzoic acid	5	26.367	22727	2469	9.470	8.203	8.49870 [ug/mL]	1.973
12	Hyperoside	5	26.883	1081	118	0.450	0.392	N/A	1.287
13	Rutin	5	27.260	75	15	0.031	0.049	N/A	0.683
14	Isoquercetin	5	27.460	1794	209	0.748	0.695	N/A	2.614
15	Resveratrol	5	28.150	15835	1677	6.598	5.570	1.91416 [ug/mL]	20.250
16	t-Cinnamic acid	5	33.933	34095	2889	14.206	9.597	N/A	4.405
17	Quercetin	5	35.793	3574	202	1.489	0.672	0.848927 [ug/mL]	31.413
18	Emodin	5	47.683	12304	1528	5.127	5.077	N/A	N/A
