# Supplementary material for: Untargeted Metabolomics Used to Describe the Chemical Composition, Antioxidant and Antimicrobial Effects of Extracts from Pleurotus spp. Mycelium Grown in Different Culture Media
Source: Antibiotics (Basel). 2022 Oct 25;11(11):1468. doi: 10.3390/antibiotics11111468 (PMC9686522; doi:10.3390/antibiotics11111468)
Supplement: Supplementary file 1 [file antibiotics-11-01468-s001.zip › S1_PE4.pdf]

Chromatogram

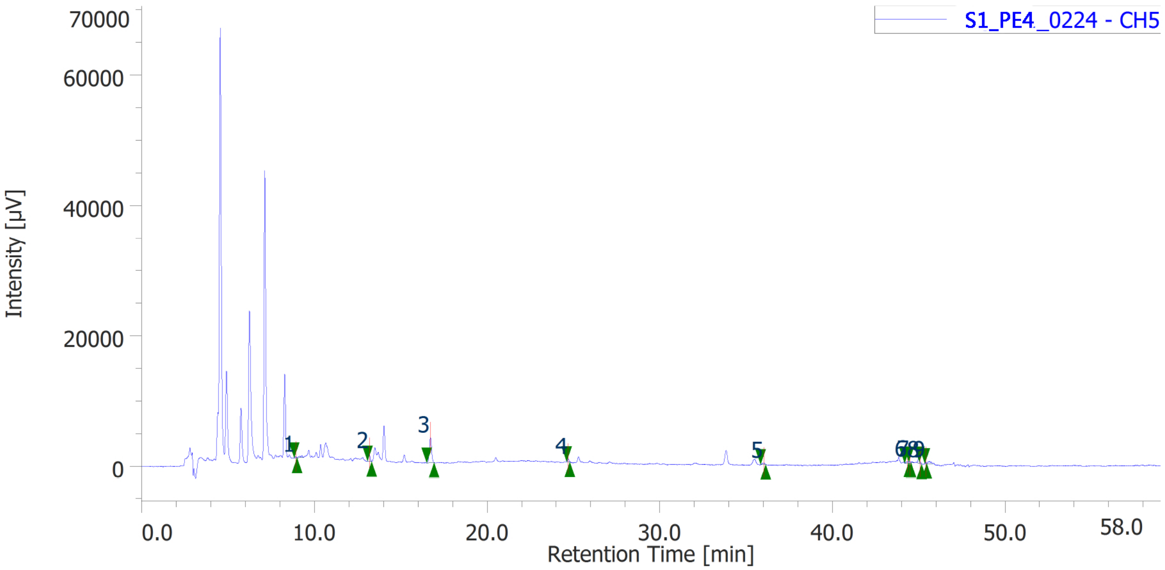

Chromatogram Information	
User Name	Administrator
Date Modified	3/15/2022 9:47:08 AM
Description	
HPLC System Name	HPLC2_DAD
Injection Date	2/26/2022 12:28:29 AM
Volume	5.00 [µL]
Sample #	21
Project Name	Phenols_08_03_2022
Acquisition Time	59.0 [min]
Acquisition Sequence	
Control Method	
Peak ID Table	PeakID_PhenolicCompound_18_02_2022_86
Calibration Method	Phenols_QUANT_CALIB_05_03_2022_Ch5-9
Additional Information	

Channel & Peak Information Table

Chromatogram Name	S1_PE4_0224-CH5
Sample Name	
Channel Name	254.0nm
Sampling Interval	200 [msec]
Peak Method	PeakMethod_05_03_2022_Extracts (Manual)

#	Peak Name	CH	tR [min]	Area [µV·sec]	Height [µV]	Quantity
1	Gallic acid	5	8.907	1260	208	N/A
2	Caftaric acid	5	13.170	7826	1256	1.41233 [ug/mL]
3	4-Hydroxybenzoic acid	5	16.717	34147	3846	N/A
4	t-Ferulic acid	5	24.693	2545	462	N/A
5	t-Cinnamic acid	5	36.013	2678	255	N/A
6	Carvacrol	5	44.340	2205	257	N/A
7	Thymol	5	44.463	1300	220	N/A
8	Flavone	5	45.070	1435	261	N/A
9	3-Hydroxyflavone	5	45.403	1005	249	N/A
