# Supplementary material for: Untargeted Metabolomics Used to Describe the Chemical Composition, Antioxidant and Antimicrobial Effects of Extracts from Pleurotus spp. Mycelium Grown in Different Culture Media
Source: Antibiotics (Basel). 2022 Oct 25;11(11):1468. doi: 10.3390/antibiotics11111468 (PMC9686522; doi:10.3390/antibiotics11111468)
Supplement: Supplementary file 1 [file antibiotics-11-01468-s001.zip › S1_PO4.pdf]

Chromatogram

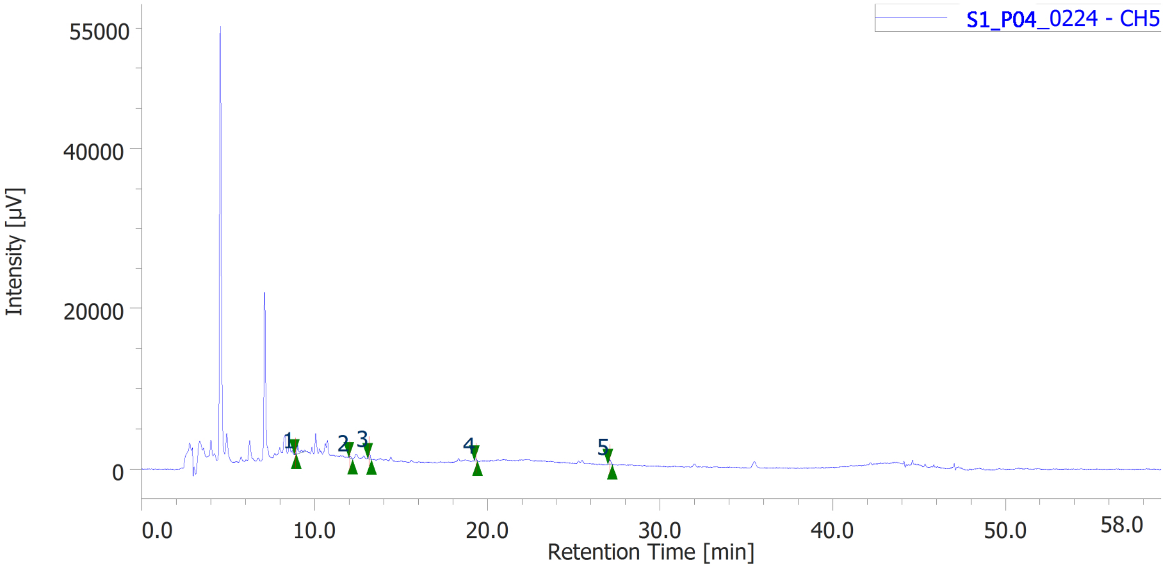

Chromatogram Information	
User Name	Administrator
Date Modified	3/14/2022 11:29:05 AM
Description	
HPLC System Name	HPLC2_DAD
Injection Date	2/25/2022 11:27:59 PM
Volume	5.00 [µL]
Sample #	20
Project Name	Phenols_08_03_2022
Acquisition Time	59.0 [min]
Acquisition Sequence	
Control Method	
Peak ID Table	PeakID_PhenolicCompound_18_02_2022_86
Calibration Method	Phenols_QUANT_CALIB_05_03_2022_Ch5-9
Additional Information	

Channel & Peak Information Table

Chromatogram Name	S1_P04_0224-CH5
Sample Name	
Channel Name	254.0nm
Sampling Interval	200 [msec]
Peak Method	PeakMethod_05_03_2022_Extracts (Manual)

#	Peak Name	CH	tR [min]	Area [µV·sec]	Height [µV]	Quantity
1	Gallic acid	5	8.897	543	129	N/A
2	3-Hydroxytyrosol	5	12.047	1987	238	0.451178 [ug/mL]
3	Caftaric acid	5	13.167	4888	863	1.15996 [ug/mL]
4	Vanillic acid	5	19.333	1812	299	N/A
5	Benzoic acid	5	27.090	4653	569	1.87404 [ug/mL]
