# Supplementary material for: Untargeted Metabolomics Used to Describe the Chemical Composition, Antioxidant and Antimicrobial Effects of Extracts from Pleurotus spp. Mycelium Grown in Different Culture Media
Source: Antibiotics (Basel). 2022 Oct 25;11(11):1468. doi: 10.3390/antibiotics11111468 (PMC9686522; doi:10.3390/antibiotics11111468)
Supplement: Supplementary file 1 [file antibiotics-11-01468-s001.zip › S1_PO5.pdf]

Chromatogram

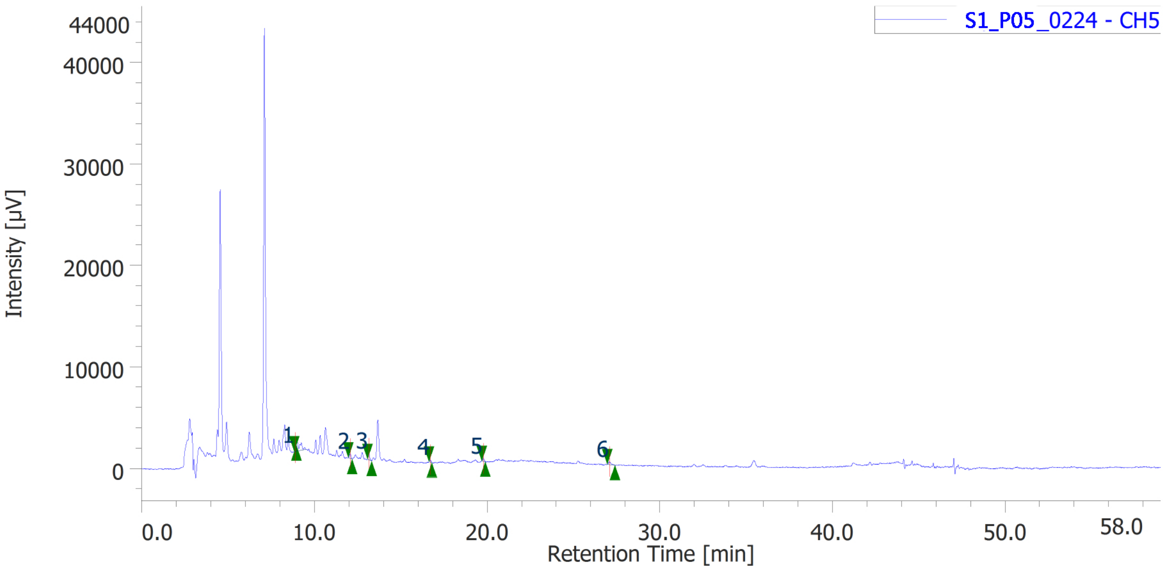

Chromatogram Information

User Name	Administrator
Date Modified	3/15/2022 9:46:00 AM
Description	
HPLC System Name	HPLC2_DAD
Injection Date	2/26/2022 1:28:58 AM
Volume	5.00 [µL]
Sample #	22
Project Name	Phenols_08_03_2022
Acquisition Time	59.0 [min]
Acquisition Sequence	
Control Method	
Peak ID Table	PeakID_PhenolicCompound_18_02_2022_86
Calibration Method	Phenols_QUANT_CALIB_05_03_2022_Ch5-9
Additional Information	

Channel & Peak Information Table

Chromatogram Name	S1_P05_0224-CH5
Sample Name	
Channel Name	254.0nm
Sampling Interval	200 [msec]
Peak Method	PeakMethod_05_03_2022_Extracts (Manual)

#	Peak Name	CH	tR [min]	Area [µV·sec]	Height [µV]	Quantity
1	Gallic acid	5	8.887	2529	359	N/A
2	3-Hydroxytyrosol	5	12.077	2913	451	0.894321 [ug/mL]
3	Caftaric acid	5	13.140	3705	611	1.05835 [ug/mL]
4	4-Hydroxybenzoic acid	5	16.697	1344	218	N/A
5	Caffeic acid	5	19.773	1642	264	N/A
6	Benzoic acid	5	27.090	2661	274	1.14383 [ug/mL]
