# Supplementary material for: Untargeted Metabolomics Used to Describe the Chemical Composition, Antioxidant and Antimicrobial Effects of Extracts from Pleurotus spp. Mycelium Grown in Different Culture Media
Source: Antibiotics (Basel). 2022 Oct 25;11(11):1468. doi: 10.3390/antibiotics11111468 (PMC9686522; doi:10.3390/antibiotics11111468)
Supplement: Supplementary file 1 [file antibiotics-11-01468-s001.zip › S1_PO6.pdf]

Chromatogram

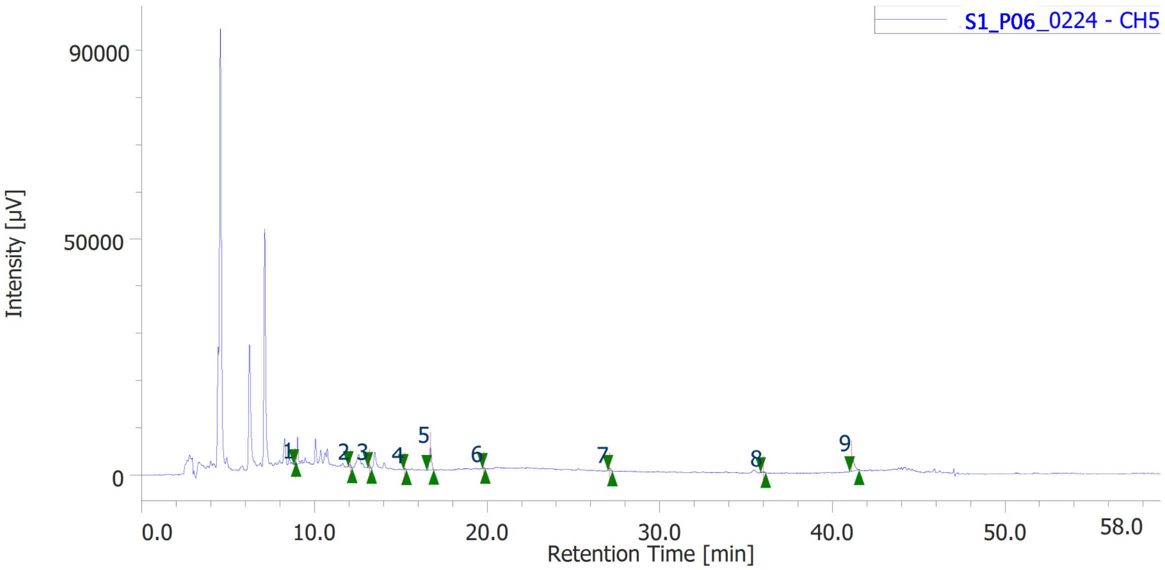

Chromatogram Information	
User Name	Administrator
Date Modified	3/15/2022 9:46:01 AM
Description	
HPLC System Name	HPLC2_DAD
Injection Date	2/26/2022 2:29:28 AM
Volume	5.00 [μL]
Sample #	23
Project Name	Phenols_08_03_2022
Acquisition Time	59.0 [min]
Acquisition Sequence	
Control Method	
Peak ID Table	PeakID_PhenolicCompound_18_02_2022_86
Calibration Method	Phenols_QUANT_CALIB_05_03_2022_Ch5-9
Additional Information	

Channel & Peak Information Table

Chromatogram Name	S1_P06_0224-CH5
Sample Name	
Channel Name	254.0nm
Sampling Interval	200 [msec]
Peak Method	PeakMethod_05_03_2022_Extracts (Manual)

#	Peak Name	CH	tR [min]	Area [μV·sec]	Height [μV]	Quantity
1	Gallic acid	5	8.877	1602	240	N/A
2	3-Hydroxytyrosol	5	12.080	4312	533	1.56411 [ug/mL]
3	Caftaric acid	5	13.173	4103	701	1.09248 [ug/mL]
4	Catechin	5	15.220	1603	275	1.86523 [ug/mL]
5	4-Hydroxybenzoic acid	5	16.713	41623	4721	N/A
6	Caffeic acid	5	19.797	1625	309	N/A
7	Benzoic acid	5	27.090	4909	583	1.96773 [ug/mL]
8	t-Cinnamic acid	5	35.983	2748	257	N/A
9	Hesperetin	5	41.120	40905	3248	11.8275 [ug/mL]
