# Supplementary material for: Untargeted Metabolomics Used to Describe the Chemical Composition, Antioxidant and Antimicrobial Effects of Extracts from Pleurotus spp. Mycelium Grown in Different Culture Media
Source: Antibiotics (Basel). 2022 Oct 25;11(11):1468. doi: 10.3390/antibiotics11111468 (PMC9686522; doi:10.3390/antibiotics11111468)
Supplement: Supplementary file 1 [file antibiotics-11-01468-s001.zip › S2_PC1.pdf]

Chromatogram

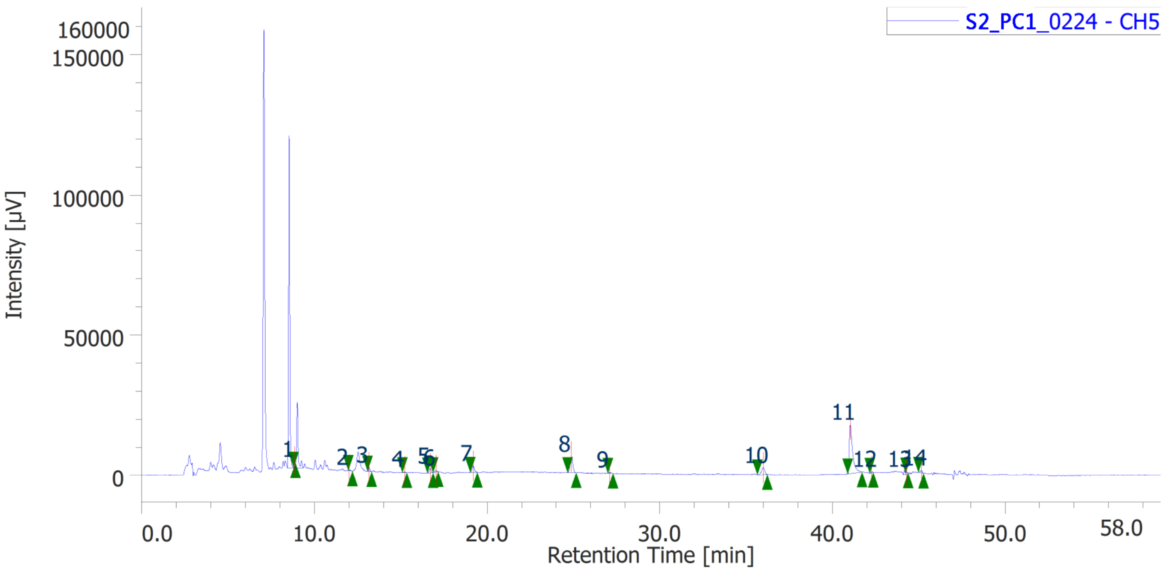

Chromatogram Information

User Name	Administrator
Date Modified	3/21/2022 10:35:59 AM
Description	
HPLC System Name	HPLC2_DAD
Injection Date	2/26/2022 3:29:57 AM
Volume	5.00 [µL]
Sample #	24
Project Name	Phenols_08_03_2022
Acquisition Time	59.0 [min]
Acquisition Sequence	
Control Method	
Peak ID Table	PeakID_PhenolicCompound_18_02_2022_86
Calibration Method	Phenols_QUANT_CALIB_05_03_2022_Ch5-9
Additional Information	

Channel & Peak Information Table

Chromatogram Name	S2_PC1_0224-CH5
Sample Name	
Channel Name	254.0nm
Sampling Interval	200 [msec]
Peak Method	PeakMethod_05_03_2022_Extracts (Manual)

#	Peak Name	CH	tR [min]	Area [µV·sec]	Height [µV]	Quantity
1	Gallic acid	5	8.863	9268	2180	N/A
2	3-Hydroxytyrosol	5	12.013	2536	350	0.713875 [ug/mL]
3	Caftaric acid	5	13.163	8097	1401	1.43557 [ug/mL]
4	Catechin	5	15.210	2470	317	2.71687 [ug/mL]
5	4-Hydroxybenzoic acid	5	16.703	13160	1556	N/A
6	Loganic acid	5	17.040	5723	574	N/A
7	Vanillic acid	5	19.187	22164	2301	N/A
8	t-Ferulic acid	5	24.880	63077	5649	3.09358 [ug/mL]
9	Benzoic acid	5	27.087	3249	374	1.35952 [ug/mL]
10	t-Cinnamic acid	5	35.973	31863	2374	N/A
11	Hesperetin	5	41.023	231862	17400	16.5416 [ug/mL]
12	Kaempferol	5	42.257	2683	420	N/A
13	Carvacrol	5	44.273	5486	852	N/A
14	Flavone	5	45.153	7627	875	N/A
