# Supplementary material for: Untargeted Metabolomics Used to Describe the Chemical Composition, Antioxidant and Antimicrobial Effects of Extracts from Pleurotus spp. Mycelium Grown in Different Culture Media
Source: Antibiotics (Basel). 2022 Oct 25;11(11):1468. doi: 10.3390/antibiotics11111468 (PMC9686522; doi:10.3390/antibiotics11111468)
Supplement: Supplementary file 1 [file antibiotics-11-01468-s001.zip › S2_PE1.pdf]

Chromatogram

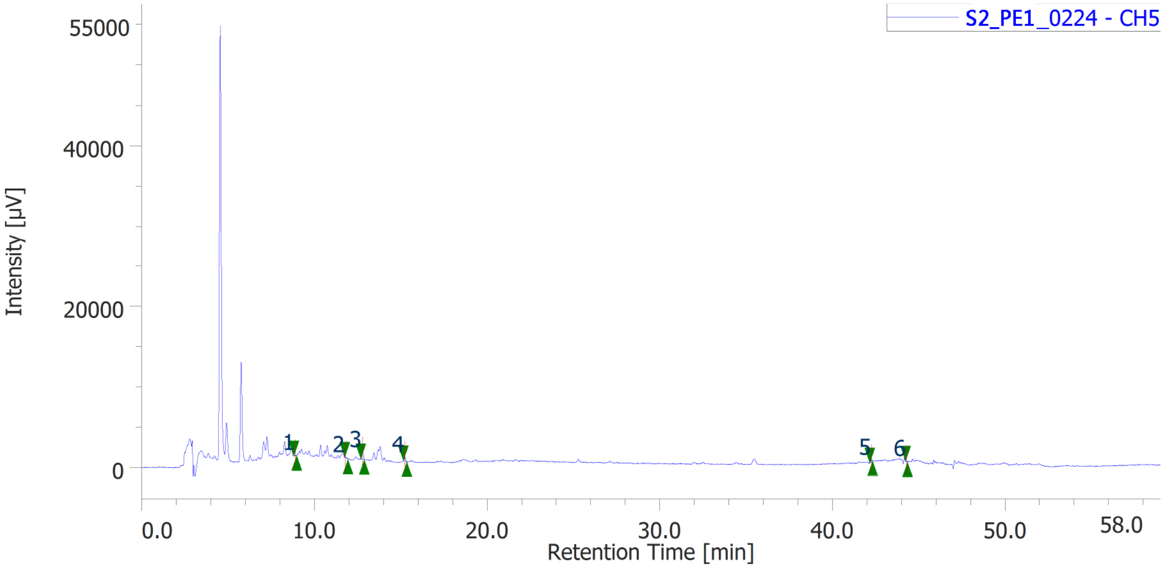

Chromatogram Information	
User Name	Administrator
Date Modified	3/21/2022 10:35:59 AM
Description	
HPLC System Name	HPLC2_DAD
Injection Date	2/26/2022 4:30:27 AM
Volume	5.00 [µL]
Sample #	25
Project Name	Phenols_08_03_2022
Acquisition Time	59.0 [min]
Acquisition Sequence	
Control Method	
Peak ID Table	PeakID_PhenolicCompound_18_02_2022_86
Calibration Method	Phenols_QUANT_CALIB_05_03_2022_Ch5-9
Additional Information	

Channel & Peak Information Table

Chromatogram Name	S2_PE1_0224-CH5
Sample Name	
Channel Name	254.0nm
Sampling Interval	200 [msec]
Peak Method	PeakMethod_05_03_2022_Extracts (Manual)

#	Peak Name	CH	tR [min]	Area [µV·sec]	Height [µV]	Quantity
1	Gallic acid	5	8.893	362	86	N/A
2	3-Hydroxytyrosol	5	11.790	583	82	N/A
3	Caftaric acid	5	12.780	5308	907	1.19602 [ug/mL]
4	Catechin	5	15.233	2919	507	3.15742 [ug/mL]
5	Kaempferol	5	42.257	1435	257	N/A
6	Carvacrol	5	44.273	313	96	N/A
