# Supplementary material for: Untargeted Metabolomics Used to Describe the Chemical Composition, Antioxidant and Antimicrobial Effects of Extracts from Pleurotus spp. Mycelium Grown in Different Culture Media
Source: Antibiotics (Basel). 2022 Oct 25;11(11):1468. doi: 10.3390/antibiotics11111468 (PMC9686522; doi:10.3390/antibiotics11111468)
Supplement: Supplementary file 1 [file antibiotics-11-01468-s001.zip › S2_PE2.pdf]

Chromatogram

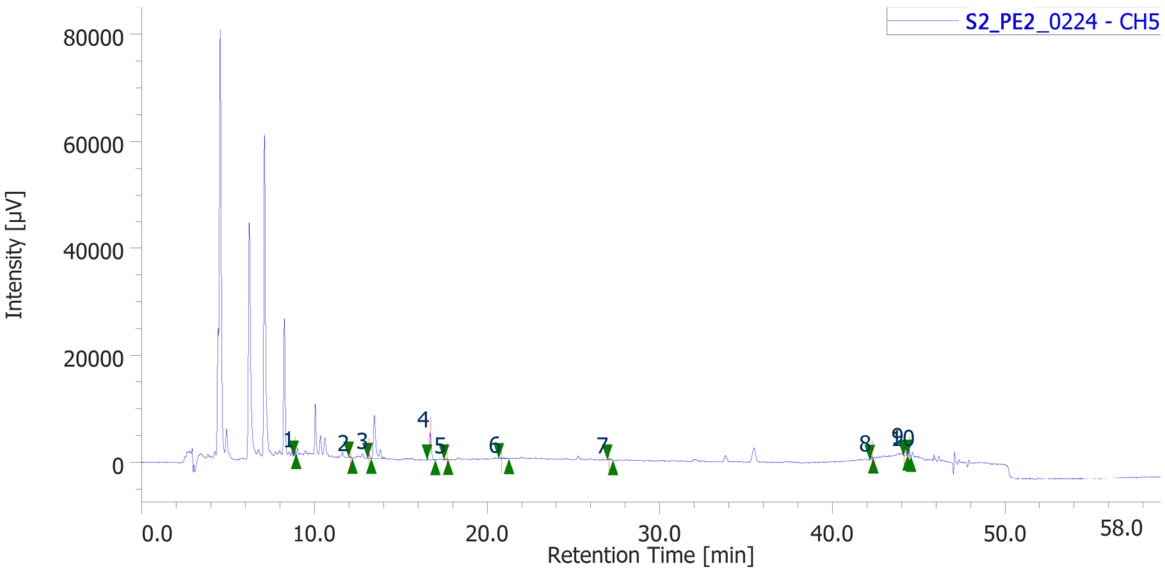

Chromatogram Information

User Name	Administrator
Date Modified	3/21/2022 10:36:00 AM
Description	
HPLC System Name	HPLC2_DAD
Injection Date	2/26/2022 8:32:25 AM
Volume	5.00 [µL]
Sample #	28
Project Name	Phenols_08_03_2022
Acquisition Time	59.0 [min]
Acquisition Sequence	
Control Method	
Peak ID Table	PeakID_PhenolicCompound_18_02_2022_86
Calibration Method	Phenols_QUANT_CALIB_05_03_2022_Ch5-9
Additional Information	

Channel & Peak Information Table

Chromatogram Name	S2_PE2_0224-CH5
Sample Name	
Channel Name	254.0nm
Sampling Interval	200 [msec]
Peak Method	PeakMethod_05_03_2022_Extracts (Manual)

#	Peak Name	CH	tR [min]	Area [µV·sec]	Height [µV]	Quantity
1	Gallic acid	5	8.893	3266	598	N/A
2	3-Hydroxytyrosol	5	12.070	1684	275	0.305922 [ug/mL]
3	Caftaric acid	5	13.157	4901	830	1.16101 [ug/mL]
4	4-Hydroxybenzoic acid	5	16.697	46545	5177	N/A
5	Chlorogenic acid	5	17.660	2110	257	N/A
6	Syringic acid	5	20.833	3362	227	N/A
7	Benzoic acid	5	27.060	2790	307	1.19114 [ug/mL]
8	Kaempferol	5	42.280	2799	422	N/A
9	Carvacrol	5	44.153	8390	1240	N/A
10	Thymol	5	44.447	4987	902	0.0454650 [ug/mL]
