# Supplementary material for: Untargeted Metabolomics Used to Describe the Chemical Composition, Antioxidant and Antimicrobial Effects of Extracts from Pleurotus spp. Mycelium Grown in Different Culture Media
Source: Antibiotics (Basel). 2022 Oct 25;11(11):1468. doi: 10.3390/antibiotics11111468 (PMC9686522; doi:10.3390/antibiotics11111468)
Supplement: Supplementary file 1 [file antibiotics-11-01468-s001.zip › S2_PE4.pdf]

Chromatogram

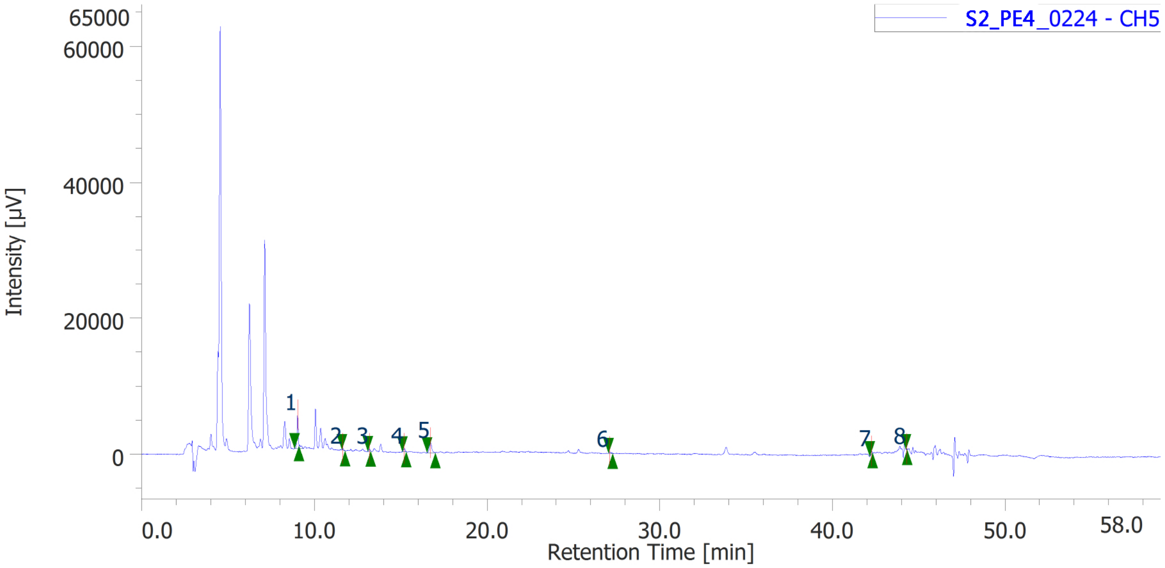

Chromatogram Information

User Name	Administrator
Date Modified	3/21/2022 10:36:01 AM
Description	
HPLC System Name	HPLC2_DAD
Injection Date	2/26/2022 2:35:19 PM
Volume	5.00 [µL]
Sample #	33
Project Name	Phenols_08_03_2022
Acquisition Time	59.0 [min]
Acquisition Sequence	
Control Method	
Peak ID Table	PeakID_PhenolicCompound_18_02_2022_86
Calibration Method	Phenols_QUANT_CALIB_05_03_2022_Ch5-9
Additional Information	

Channel & Peak Information Table

Chromatogram Name	S2_PE4_0224-CH5
Sample Name	
Channel Name	254.0nm
Sampling Interval	200 [msec]
Peak Method	PeakMethod_05_03_2022_Extracts (Manual)

#	Peak Name	CH	tR [min]	Area [µV·sec]	Height [µV]	Quantity
1	Gallic acid	5	9.023	24885	4901	0.951539 [ug/mL]
2	3-Hydroxytyrosol	5	11.640	1116	222	0.0341761 [ug/mL]
3	Caftaric acid	5	13.177	1967	394	0.908978 [ug/mL]
4	Catechin	5	15.183	3259	484	3.49181 [ug/mL]
5	4-Hydroxybenzoic acid	5	16.713	14429	1483	N/A
6	Benzoic acid	5	27.090	1571	200	0.744453 [ug/mL]
7	Kaempferol	5	42.267	2666	465	N/A
8	Carvacrol	5	44.280	183	91	N/A
