# Supplementary material for: Untargeted Metabolomics Used to Describe the Chemical Composition, Antioxidant and Antimicrobial Effects of Extracts from Pleurotus spp. Mycelium Grown in Different Culture Media
Source: Antibiotics (Basel). 2022 Oct 25;11(11):1468. doi: 10.3390/antibiotics11111468 (PMC9686522; doi:10.3390/antibiotics11111468)
Supplement: Supplementary file 1 [file antibiotics-11-01468-s001.zip › S2_PN.pdf]

# Chromatogram

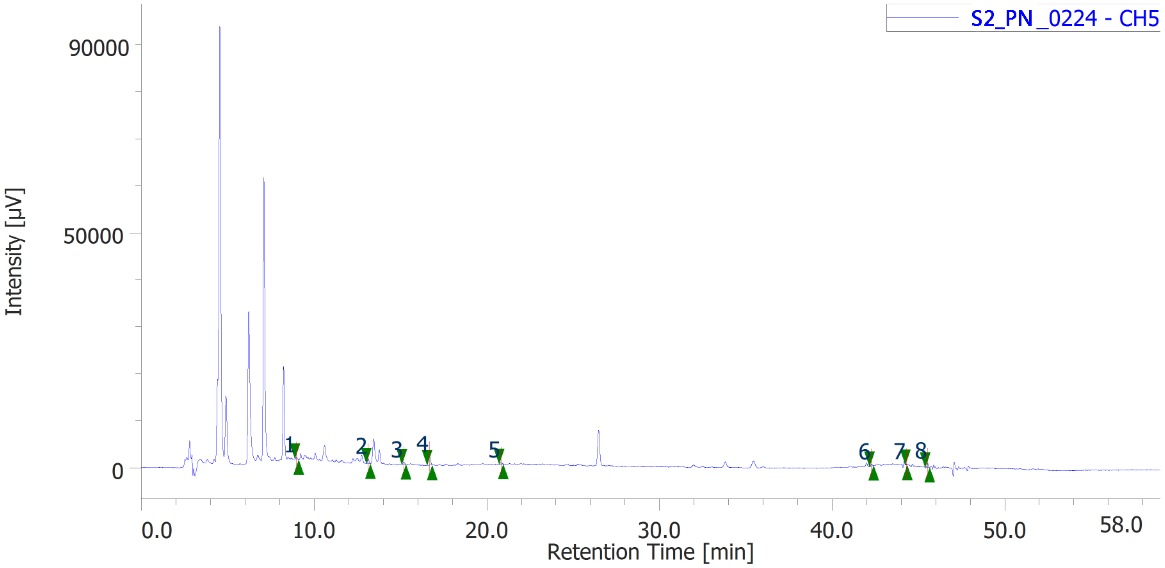


| | |
|---|---|
| Chromatogram Information | |
| User Name | Administrator |
| Date Modified | 3/21/2022 10:35:59 AM |
| Description | |
| HPLC System Name | HPLC2_DAD |
| Injection Date | 2/26/2022 7:31:55 AM |
| Volume | 5.00 [µL] |
| Sample # | 27 |
| Project Name | Phenols_08_03_2022 |
| Acquisition Time | 59.0 [min] |
| Acquisition Sequence | |
| Control Method | |
| Peak ID Table | PeakID_PhenolicCompound_18_02_2022_86 |
| Calibration Method | Phenols_QUANT_CALIB_05_03_2022_Ch5-9 |
| Additional Information | |

**Channel & Peak Information Table**

| | |
|---|---|
| Chromatogram Name | S2_PN_0224-CH5 |
| Sample Name | |
| Channel Name | 254.0nm |
| Sampling Interval | 200 [msec] |
| Peak Method | PeakMethod_05_03_2022_Extracts (Manual) |

| # | Peak Name | CH | tR [min] | Area [µV·sec] | Height [µV] | Quantity |
|---|---|---|---|---|---|---|
| 1 | Gallic acid | 5 | 8.977 | 2151 | 372 | N/A |
| 2 | Caftaric acid | 5 | 13.127 | 6124 | 1013 | 1.26610 [ug/mL] |
| 3 | Catechin | 5 | 15.183 | 3259 | 441 | 3.49210 [ug/mL] |
| 4 | 4-Hydroxybenzoic acid | 5 | 16.670 | 14398 | 1706 | N/A |
| 5 | Syringic acid | 5 | 20.807 | 2479 | 337 | N/A |
| 6 | Kaempferol | 5 | 42.263 | 2795 | 403 | N/A |
| 7 | Carvacrol | 5 | 44.273 | 579 | 180 | N/A |
| 8 | 3-Hydroxyflavone | 5 | 45.520 | 4768 | 899 | N/A |
