# Supplementary material for: Untargeted Metabolomics Used to Describe the Chemical Composition, Antioxidant and Antimicrobial Effects of Extracts from Pleurotus spp. Mycelium Grown in Different Culture Media
Source: Antibiotics (Basel). 2022 Oct 25;11(11):1468. doi: 10.3390/antibiotics11111468 (PMC9686522; doi:10.3390/antibiotics11111468)
Supplement: Supplementary file 1 [file antibiotics-11-01468-s001.zip › S2_PO1.pdf]

Chromatogram

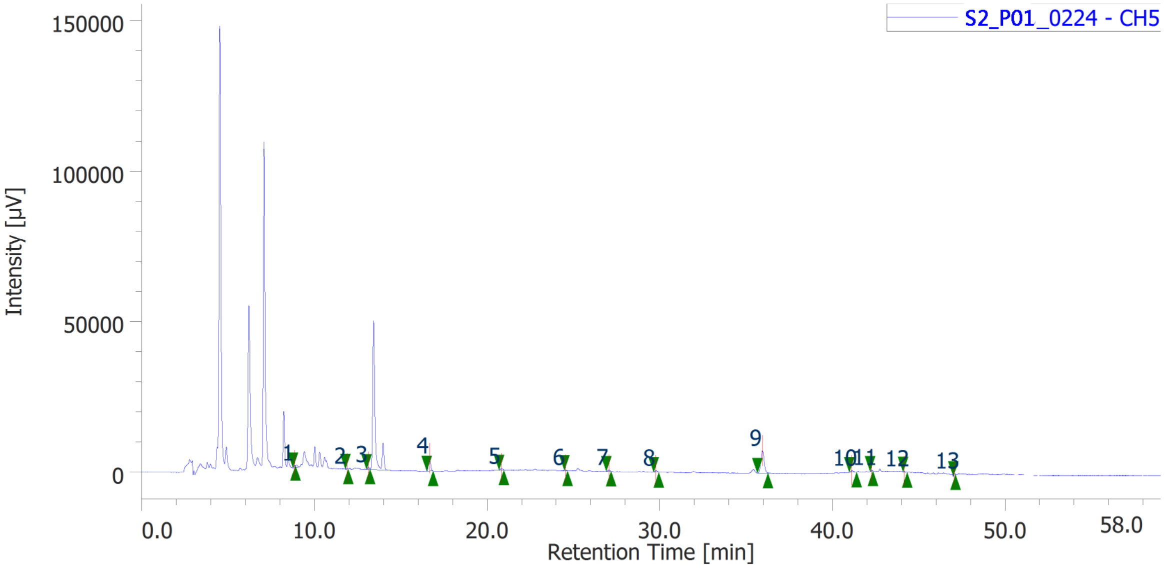

Chromatogram Information

User Name	Administrator
Date Modified	3/21/2022 10:35:59 AM
Description	
HPLC System Name	HPLC2_DAD
Injection Date	2/26/2022 6:31:26 AM
Volume	5.00 [μL]
Sample #	26
Project Name	Phenols_08_03_2022
Acquisition Time	59.0 [min]
Acquisition Sequence	
Control Method	
Peak ID Table	PeakID_PhenolicCompound_18_02_2022_86
Calibration Method	Phenols_QUANT_CALIB_05_03_2022_Ch5-9
Additional Information	

Channel & Peak Information Table

Chromatogram Name	S2_P01_0224-CH5
Sample Name	
Channel Name	254.0nm
Sampling Interval	200 [msec]
Peak Method	PeakMethod_05_03_2022_Extracts (Manual)

#	Peak Name	CH	tR [min]	Area [μV·sec]	Height [μV]	Quantity
1	Gallic acid	5	8.863	3673	754	N/A
2	3-Hydroxytyrosol	5	11.873	726	152	N/A
3	Caftaric acid	5	13.123	5261	886	1.19200 [ug/mL]
4	4-Hydroxybenzoic acid	5	16.663	36260	4166	N/A
5	Syringic acid	5	20.823	4948	529	N/A
6	t-Ferulic acid	5	24.543	1276	208	N/A
7	Benzoic acid	5	27.057	4107	506	1.67374 [ug/mL]
8	Rosmarinic acid	5	29.777	5920	640	N/A
9	t-Cinnamic acid	5	35.940	101844	7536	1.33915 [ug/mL]
10	Hesperetin	5	41.110	6719	606	2.03236 [ug/mL]
11	Kaempferol	5	42.267	2479	477	N/A
12	Carvacrol	5	44.143	2056	356	N/A
13	Emodin	5	47.053	3336	807	N/A
