# Supplementary material for: Untargeted Metabolomics Used to Describe the Chemical Composition, Antioxidant and Antimicrobial Effects of Extracts from Pleurotus spp. Mycelium Grown in Different Culture Media
Source: Antibiotics (Basel). 2022 Oct 25;11(11):1468. doi: 10.3390/antibiotics11111468 (PMC9686522; doi:10.3390/antibiotics11111468)
Supplement: Supplementary file 1 [file antibiotics-11-01468-s001.zip › S2_PO2.pdf]

Chromatogram

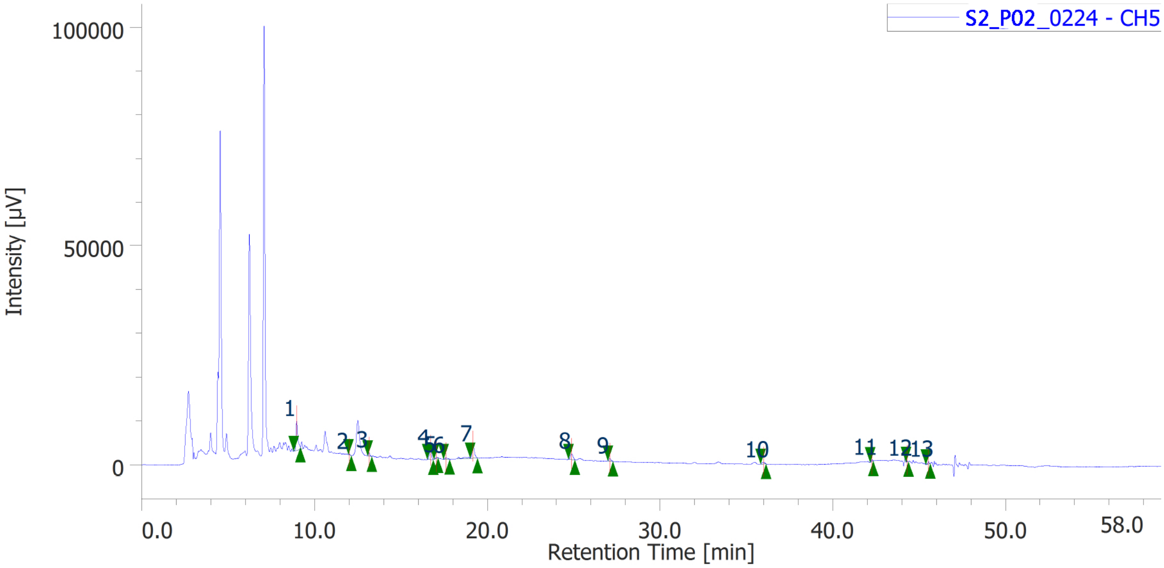

Chromatogram Information

User Name	Administrator
Date Modified	3/21/2022 10:36:00 AM
Description	
HPLC System Name	HPLC2_DAD
Injection Date	2/26/2022 10:33:24 AM
Volume	5.00 [µL]
Sample #	30
Project Name	Phenols_08_03_2022
Acquisition Time	59.0 [min]
Acquisition Sequence	
Control Method	
Peak ID Table	PeakID_PhenolicCompound_18_02_2022_86
Calibration Method	Phenols_QUANT_CALIB_05_03_2022_Ch5-9
Additional Information	

Channel & Peak Information Table

Chromatogram Name	S2_PO2_0224-CH5
Sample Name	
Channel Name	254.0nm
Sampling Interval	200 [msec]
Peak Method	PeakMethod_05_03_2022_Extracts (Manual)

#	Peak Name	CH	tR [min]	Area [µV·sec]	Height [µV]	Quantity
1	Gallic acid	5	8.960	49797	6701	2.53289 [ug/mL]
2	3-Hydroxytyrosol	5	12.013	1107	222	0.0297715 [ug/mL]
3	Caftaric acid	5	13.150	4471	793	1.12407 [ug/mL]
4	4-Hydroxybenzoic acid	5	16.697	17645	2059	N/A
5	Loganic acid	5	17.060	2617	311	N/A
6	Chlorogenic acid	5	17.583	4160	340	0.110922 [ug/mL]
7	Vanillic acid	5	19.173	26247	2688	N/A
8	t-Ferulic acid	5	24.877	14151	1326	0.181858 [ug/mL]
9	Benzoic acid	5	27.073	4747	553	1.90835 [ug/mL]
10	t-Cinnamic acid	5	35.997	4315	378	N/A
11	Kaempferol	5	42.260	2665	442	N/A
12	Carvacrol	5	44.280	1202	313	N/A
13	3-Hydroxyflavone	5	45.527	3337	587	N/A
