# Supplementary material for: Untargeted Metabolomics Used to Describe the Chemical Composition, Antioxidant and Antimicrobial Effects of Extracts from Pleurotus spp. Mycelium Grown in Different Culture Media
Source: Antibiotics (Basel). 2022 Oct 25;11(11):1468. doi: 10.3390/antibiotics11111468 (PMC9686522; doi:10.3390/antibiotics11111468)
Supplement: Supplementary file 1 [file antibiotics-11-01468-s001.zip › S2_PO3.pdf]

Chromatogram

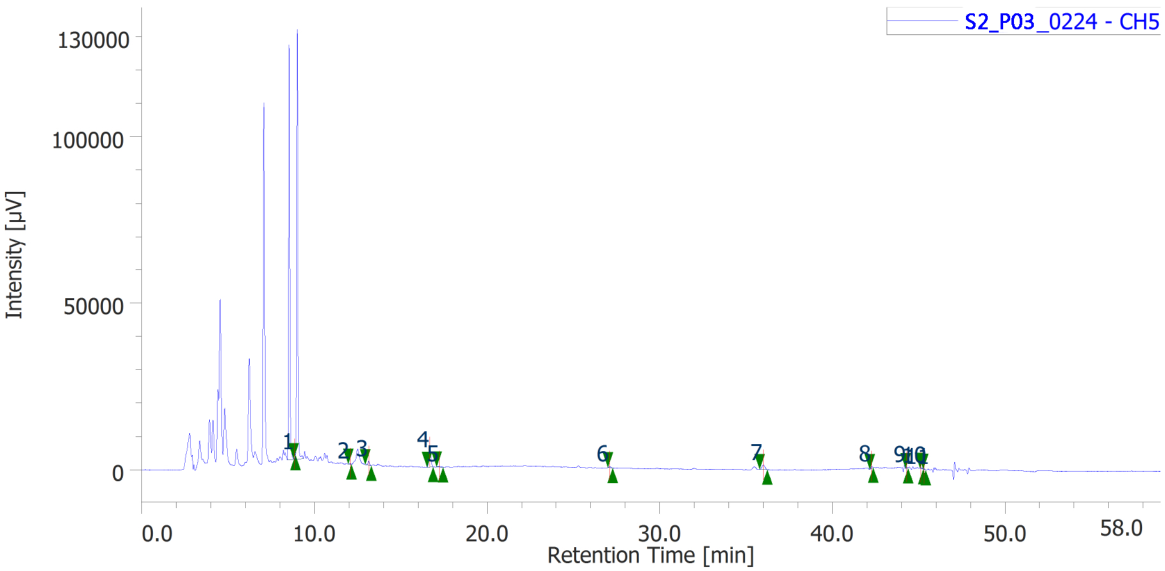

Chromatogram Information

User Name	Administrator
Date Modified	3/21/2022 10:36:00 AM
Description	
HPLC System Name	HPLC2_DAD
Injection Date	2/26/2022 11:33:53 AM
Volume	5.00 [µL]
Sample #	31
Project Name	Phenols_08_03_2022
Acquisition Time	59.0 [min]
Acquisition Sequence	
Control Method	
Peak ID Table	PeakID_PhenolicCompound_18_02_2022_86
Calibration Method	Phenols_QUANT_CALIB_05_03_2022_Ch5-9
Additional Information	

Channel & Peak Information Table

Chromatogram Name	S2_P03_0224-CH5
Sample Name	
Channel Name	254.0nm
Sampling Interval	200 [msec]
Peak Method	PeakMethod_05_03_2022_Extracts (Manual)

#	Peak Name	CH	tR [min]	Area [µV·sec]	Height [µV]	Quantity
1	Gallic acid	5	8.860	6620	1581	N/A
2	3-Hydroxytyrosol	5	12.060	1359	264	0.150420 [ug/mL]
3	Caftaric acid	5	13.140	8253	1268	1.44894 [ug/mL]
4	4-Hydroxybenzoic acid	5	16.687	39072	4471	N/A
5	Loganic acid	5	17.237	2465	205	N/A
6	Benzoic acid	5	27.090	3230	401	1.35241 [ug/mL]
7	t-Cinnamic acid	5	35.987	16768	1353	N/A
8	Kaempferol	5	42.267	3704	608	N/A
9	Carvacrol	5	44.283	1805	456	N/A
10	Flavone	5	45.117	1817	279	N/A
11	3-Hydroxyflavone	5	45.303	1008	234	N/A
