# Supplementary material for: Untargeted Metabolomics Used to Describe the Chemical Composition, Antioxidant and Antimicrobial Effects of Extracts from Pleurotus spp. Mycelium Grown in Different Culture Media
Source: Antibiotics (Basel). 2022 Oct 25;11(11):1468. doi: 10.3390/antibiotics11111468 (PMC9686522; doi:10.3390/antibiotics11111468)
Supplement: Supplementary file 1 [file antibiotics-11-01468-s001.zip › S2_PO4.pdf]

Chromatogram

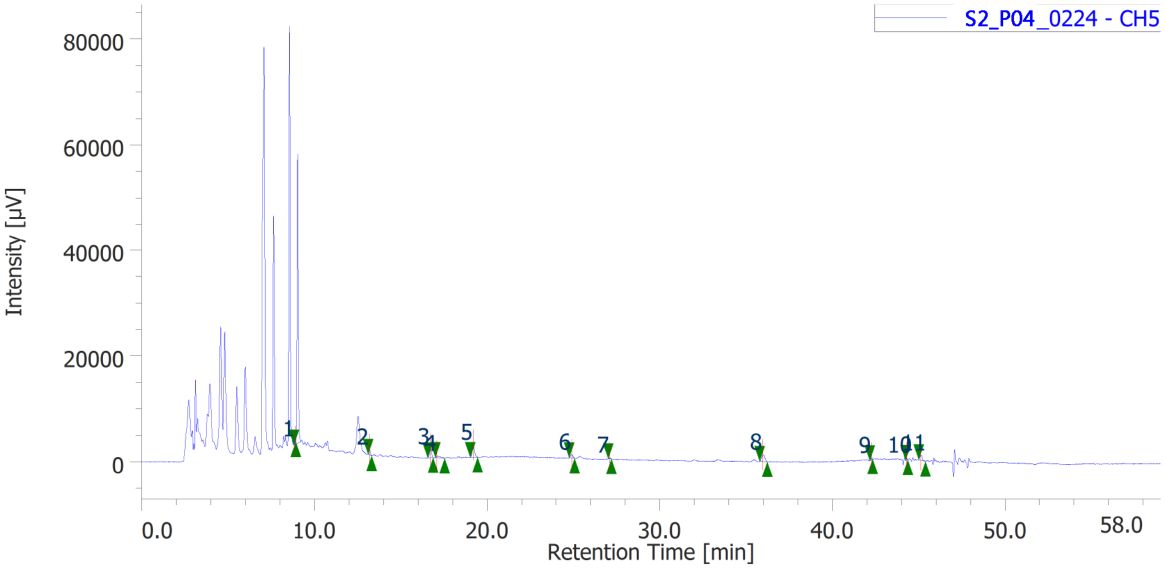

Chromatogram Information

User Name	Administrator
Date Modified	3/21/2022 10:36:01 AM
Description	
HPLC System Name	HPLC2_DAD
Injection Date	2/26/2022 1:34:50 PM
Volume	5.00 [µL]
Sample #	32
Project Name	Phenols_08_03_2022
Acquisition Time	59.0 [min]
Acquisition Sequence	
Control Method	
Peak ID Table	PeakID_PhenolicCompound_18_02_2022_86
Calibration Method	Phenols_QUANT_CALIB_05_03_2022_Ch5-9
Additional Information	

Channel & Peak Information Table

Chromatogram Name	S2_P04_0224-CH5
Sample Name	
Channel Name	254.0nm
Sampling Interval	200 [msec]
Peak Method	PeakMethod_05_03_2022_Extracts (Manual)

#	Peak Name	CH	tR [min]	Area [µV·sec]	Height [µV]	Quantity
1	Gallic acid	5	8.893	1960	560	N/A
2	Caftaric acid	5	13.180	5435	993	1.20690 [ug/mL]
3	4-Hydroxybenzoic acid	5	16.713	13309	1599	N/A
4	Loganic acid	5	17.097	5500	349	N/A
5	Vanillic acid	5	19.197	22165	2365	N/A
6	t-Ferulic acid	5	24.890	6368	643	N/A
7	Benzoic acid	5	27.097	1512	224	0.722938 [ug/mL]
8	t-Cinnamic acid	5	35.960	16870	1313	N/A
9	Kaempferol	5	42.260	1660	306	N/A
10	Carvacrol	5	44.280	1881	486	N/A
11	Flavone	5	45.097	9898	906	N/A
