# Supplementary material for: Untargeted Metabolomics Used to Describe the Chemical Composition, Antioxidant and Antimicrobial Effects of Extracts from Pleurotus spp. Mycelium Grown in Different Culture Media
Source: Antibiotics (Basel). 2022 Oct 25;11(11):1468. doi: 10.3390/antibiotics11111468 (PMC9686522; doi:10.3390/antibiotics11111468)
Supplement: Supplementary file 1 [file antibiotics-11-01468-s001.zip › S2_PO5.pdf]

Chromatogram

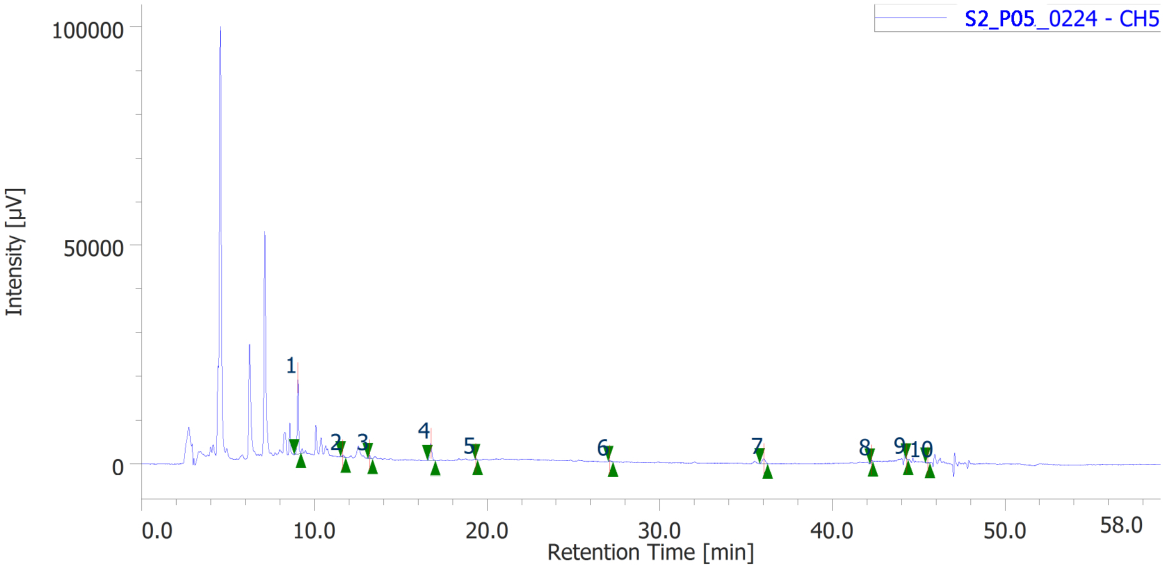

Chromatogram Information	
User Name	Administrator
Date Modified	3/21/2022 10:36:01 AM
Description	
HPLC System Name	HPLC2_DAD
Injection Date	2/26/2022 3:35:49 PM
Volume	5.00 [µL]
Sample #	34
Project Name	Phenols_08_03_2022
Acquisition Time	59.0 [min]
Acquisition Sequence	
Control Method	
Peak ID Table	PeakID_PhenolicCompound_18_02_2022_86
Calibration Method	Phenols_QUANT_CALIB_05_03_2022_Ch5-9
Additional Information	

Channel & Peak Information Table

Chromatogram Name	S2_P05_0224-CH5
Sample Name	
Channel Name	254.0nm
Sampling Interval	200 [msec]
Peak Method	PeakMethod_05_03_2022_Extracts (Manual)

#	Peak Name	CH	tR [min]	Area [µV·sec]	Height [µV]	Quantity
1	Gallic acid	5	9.043	91947	17416	5.20839 [ug/mL]
2	3-Hydroxytyrosol	5	11.647	3303	452	1.08113 [ug/mL]
3	Caftaric acid	5	13.193	4751	804	1.14813 [ug/mL]
4	4-Hydroxybenzoic acid	5	16.730	34352	3878	N/A
5	Vanillic acid	5	19.360	1147	218	N/A
6	Benzoic acid	5	27.097	2209	271	0.978112 [ug/mL]
7	t-Cinnamic acid	5	36.007	14266	1094	N/A
8	Kaempferol	5	42.267	3301	560	N/A
9	Carvacrol	5	44.273	372	137	N/A
10	3-Hydroxyflavone	5	45.520	1335	170	N/A
