# Supplementary material for: Untargeted Metabolomics Used to Describe the Chemical Composition, Antioxidant and Antimicrobial Effects of Extracts from Pleurotus spp. Mycelium Grown in Different Culture Media
Source: Antibiotics (Basel). 2022 Oct 25;11(11):1468. doi: 10.3390/antibiotics11111468 (PMC9686522; doi:10.3390/antibiotics11111468)
Supplement: Supplementary file 1 [file antibiotics-11-01468-s001.zip › S2_PO6.pdf]

Chromatogram

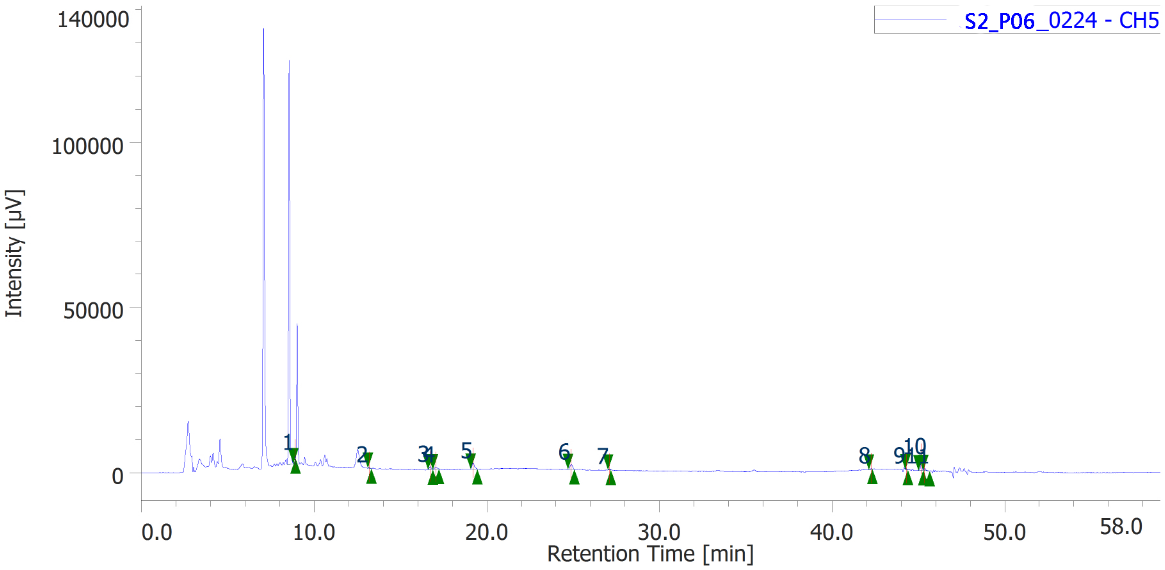

Chromatogram Information	
User Name	Administrator
Date Modified	3/21/2022 10:36:01 AM
Description	
HPLC System Name	HPLC2_DAD
Injection Date	2/26/2022 4:36:18 PM
Volume	5.00 [µL]
Sample #	35
Project Name	Phenols_08_03_2022
Acquisition Time	59.0 [min]
Acquisition Sequence	
Control Method	
Peak ID Table	PeakID_PhenolicCompound_18_02_2022_86
Calibration Method	Phenols_QUANT_CALIB_05_03_2022_Ch5-9
Additional Information	

Channel & Peak Information Table

Chromatogram Name	S2_P06_0224-CH5
Sample Name	
Channel Name	254.0nm
Sampling Interval	200 [msec]
Peak Method	PeakMethod_05_03_2022_Extracts (Manual)

#	Peak Name	CH	tR [min]	Area [µV·sec]	Height [µV]	Quantity
1	Gallic acid	5	8.873	13615	2825	0.236166 [ug/mL]
2	Caftaric acid	5	13.173	1724	319	0.888139 [ug/mL]
3	4-Hydroxybenzoic acid	5	16.713	7005	898	N/A
4	Loganic acid	5	17.027	6578	619	0.0359548 [ug/mL]
5	Vanillic acid	5	19.200	18167	1810	N/A
6	t-Ferulic acid	5	24.883	15311	1439	0.250916 [ug/mL]
7	Benzoic acid	5	27.097	1703	277	0.792797 [ug/mL]
8	Kaempferol	5	42.267	1332	217	N/A
9	Carvacrol	5	44.273	1595	405	N/A
10	Flavone	5	45.160	17779	3381	N/A
11	3-Hydroxyflavone	5	45.343	4974	706	N/A
