# Supplementary material for: Untargeted Metabolomics Used to Describe the Chemical Composition, Antioxidant and Antimicrobial Effects of Extracts from Pleurotus spp. Mycelium Grown in Different Culture Media
Source: Antibiotics (Basel). 2022 Oct 25;11(11):1468. doi: 10.3390/antibiotics11111468 (PMC9686522; doi:10.3390/antibiotics11111468)
Supplement: Supplementary file 1 [file antibiotics-11-01468-s001.zip › Supplementary MaterialsREV2.pdf]

Table S1. Preparation of mycelium extracts obtained from medium S1.

	Lyophilised mycelia weight (g)	Final concentration (mg ml^{-1})
**Sample ID**		
**PC1**	0.0375	10
**PE1**	0.0179	10
**PO1**	0.0232	10
**PN**	0.0434	10
**PE2**	0.0222	10
**PE3**	0.0326	10
**PO2**	0.0423	10
**PO3**	0.0407	10
**PO4**	0.0415	10
**PE4**	0.0573	10
**PO5**	0.0503	10
**PO6**	0.042	10

Table S2. Preparation of mycelium extracts obtained from medium S2.

	Lyophilised mycelia weight (g)	Final concentration (mg ml^{-1})
**Sample ID**		
**PC1**	0.0654	10
**PE1**	0.0813	10
**PO1**	0.028	10
**PN**	0.0419	10
**PE2**	0.0268	10
**PE3**	0.0328	10
**PO2**	0.0343	10
**PO3**	0.0387	10
**PO4**	0.0347	10
**PE4**	0.1036	10
**PO5**	0.0457	10
**PO6**	0.0321	10

Peak Name	S1_PC1	S1_PE1	S1_PO1	S1_PN	S1_PE2	S1_PE3	S1_PO2	S1_PO3	S1_PO4	S1_PE4	S1_PO5	S1_PO6	S2_PC1	S2_PE1	S2_PO1	S2_PN	S2_PE2	S2_PE3	S2_PO2	S2_PO3	S2_PO4	S2_PE4	S2_PO5	S2_PO6
**Gallic acid**	0	3.70209	0.146672	0	6.17875	0	0		0	0	0	0	0	0	0	0	0	0	2.53289	0	0	0.951539	5.20839	0.236166
**3-Hydroxytyrosol**	0	2.01849		0	2.35742			0	0.451178		0.894321	1.56411	0.713875	0	0		0.305922	0.263805	0.0297715	0.15042		0.0341761	1.08113	
**Caftaric acid**	1.13593	1.01208	1.0346	1.51619	0.944234	1.21918	0.865307	1.47035	1.15996	1.41233	1.05835	1.09248	0.43557	1.19602	1.192	1.2661	1.16101	1.16567	1.12407	1.44894	1.2069	0.908978	1.14813	0.888139
**Catechin**		4.55133			4.09636							1.86523	2.71668	3.15742		3.4921						3.49181		
**4-Hydroxybenzoic acid**	0	0.161037	0	0	0		0	0		0	0	0	0		0	0	0	0	0	0	0	0	0	0
**Loganic acid**		0											0						0	0	0			0.0359548
**Chlorogenic acid**																	0	0	0.110922					
**Vanillic acid**	0	0			0		0		0				0						0		0		0	0
**Caffeic acid**											0	0												
**Epicatechin**		9.48965			0																			
**Syringic acid**		0			0										0	0	0	0						
**p-Coumaric acid**		0			0																			
**t-Ferulic acid**		0.584954			0					0			3.09358		0				0.181858		0			0.250916
**Benzoic acid**	0.81072			0.786383	8.4987	0.494009	1.24531	3.61019	1.87404		1.14383	1.96773	1.35952		1.67374		1.19114		1.90835	1.35241	0.722938		0.978112	0.792797
**Hyperoside**					0																			
**Rutin**					0																			
**Isoquercetin**					0																			
**Resveratrol**					1.91416																			
**Rosmarinic acid**															0									
**t-Cinnamic acid**	0.4082235	0	0		0			0		0		0	0		1.33915				0	0	0		0	
**Quercetin**		0.833611			0.848927																			
**Hesperetin**												11.8275	16.5416		2.03236									
**Kaempferol**						0							0	0	0	0	0	0	0	0	0		0	0
**Carvacrol**	0		0	0						0			0	0	0	0	0		0	0	0		0	0
**Thymol**	0			0				0		0							0.015465	0.0354455						
**Flavone**	0									0			0					0		0	0			0
**3-Hydroxyflavone**	0		0	0		0				0						0		0	0	0			0	0
**Emodin**		0			0										0									

Table S3. Quantitative analysis of phenolic compounds in the extracts.
